# Effects of nitrate limitation on the metabolome of *Tetraselmis suecica* biofilms

**DOI:** 10.1016/j.crmicr.2025.100501

**Published:** 2025-10-30

**Authors:** Julien Lopez, Amélie Talec, Stéphane Greff, Andrea Fanesi, Beat Gasser, Emna Krichen, Olivier Bernard, Antoine Sciandra

**Affiliations:** a Sorbonne Université, CNRS, Laboratoire d'Océanographie de Villefranche, LOV, 06230 Villefranche-sur-Mer, France; b Aix Marseille Université, Avignon Université, CNRS, IRD, IMBE, Station Marine d’Endoume, Chemin de la Batterie des lions, 13007 Marseille, France; c Laboratoire Génie des Procédés et Matériaux (LGPM), CentraleSupélec, Université Paris-Saclay, 91190 Gif-sur-Yvette, France; d International Atomic Energy Agency (IAEA), Environment Laboratories, MC98000, Monaco; e Greenowl, INRIA, Université Côte d’Azur, 06902 Sophia Antipolis Cedex, France

**Keywords:** Phototrophic biofilm, *Tetraselmis suecica*, Nitrogen, Metabolomics, Nitric oxide

## Abstract

Phototrophic biofilms are photosynthetic microbial communities adhered to submerged surfaces. Research has largely focused on multispecies periphyton and benthic diatoms, while Chlorophyte-based, monospecific biofilms remain understudied – despite their increasing industrial relevance, particularly for the production of high value compounds. Here, we investigate the impact of nitrogen limitation on the metabolome of the green microalga *Tetraselmis suecica* grown in nitrogen-replete and nitrogen-limited monospecific biofilms. A specific culture system was developed to optimise the analysis of both the entire biofilm metabolome and spatial biochemical variations across cell layers. The Droop model was used to determine optimal initial conditions and sampling times. Then, metabolomic analysis by UHPLC-ESI(+)-QToF-HRMS/MS coupled with complementary biochemical analyses was performed on both conditions. Compared to nitrogen-replete biofilms, nitrogen-limited biofilms exhibited elevated C:N ratios (+277.4 %), reduced photosynthetic activity, and decreased pigment content (-18 % for Chl *a* and *b*). While total biovolume remained similar between experimental conditions, nitrogen limitation led to a redistribution of cell biomass, with increased surface layer biovolume (+112.3 %) at the expense of deeper layers. Macromolecular ratios of carbohydrates/proteins and lipids/proteins increased two- to three-fold, respectively, under nitrogen-limited conditions. The characterized metabolomic profile was dominated by monogalactosyldiacylglycerols (MGDGs) and digalactosyldiacylglycerols, whose relative abundances were significantly higher in nitrogen-replete condition. Notably, the annotated lipid MGDG(18:3/16:4) previously exhibited nitric oxide inhibitory activity. Given the previously observed role of nitric oxide in bacterial biofilm formation and diatom adhesion, we hypothesize that a feedback loop-like mechanism of adhesion regulation dependent on nitric oxide and nitrogen environmental conditions exists in monospecific phototrophic biofilms.

## Highlights

• A single-species phototrophic biofilm culture system in Erlenmeyer flasks was created.
• The metabolome of *Tetraselmis suecica* biofilm under nitrate limitation was analysed.
• Relative carbohydrate and lipid contents increased under nitrogen limitation.
• Some galactosyldiacylglycerols are mainly expressed under nitrogen-rich conditions.
• A nitric oxide-mediated adhesion control mechanism similar to bacteria is suspected.

## Graphical abstract

**Figure:**
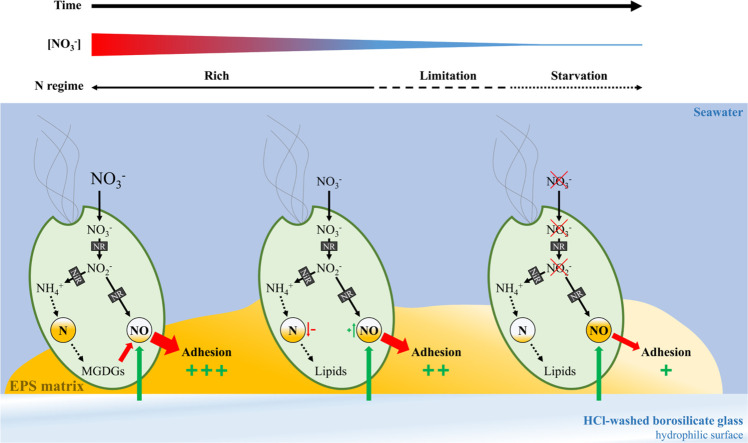

## 1 Introduction

Biofilms are microbial communities attached to a matrix of self-produced extracellular polymeric substances (EPS) and adhered to a humid surface (Flemming and Wingender, 2010). Among them, microalgal biofilms have promising applications, such as wastewater treatment (Wang and Hong, 2022), agriculture (Bharti et al., 2017), valuable products (Pierre et al., 2019), or greenhouse gas sequestration (Ugya et al., 2023). Their development is influenced by a wide range of environmental factors (Katarzyna et al., 2015). In bacterial biofilms, nutrient concentrations are known to impact both their three-dimensional structure and metabolome (Cherifi et al., 2017; Carriot et al., 2022; de Carvalho Silvello et al., 2022). In contrast, studies on monospecific phototrophic biofilms – in particular those formed by green algae – and, by extension, their production of compounds of interest, remain limited. Omics techniques are high-throughput methods for studying various classes of biological molecules (Manzoni et al., 2018). Among these, metabolomics allows the comprehensive profiling of the metabolome, i.e. all metabolites produced by cellular activity, providing an in-depth understanding of the functioning of biofilms (Vohra et al., 2024).

Nitrogen (N) is a nutrient and constituent element of microalgae, whose limitation affects both growth and metabolic production. N limitation generally results in a reorientation of the metabolism associated with protein synthesis towards carbohydrate and/or lipid synthesis (Markou et al., 2012; Yaakob et al., 2021). Various studies have shown that EPS or other molecules of interest produced in microalgal biofilm are sensitive to the source and concentration of N in the medium (Sun et al., 2024; González et al., 2020; Li et al., 2024; Cheng et al., 2013). For example, green algae *Botryococcus braunii* and *Chlorella* sp. under N deficiency have promoted their EPS production (Boonchai et al., 2015; Shen et al., 2015), while the latter is stimulated by increasing concentration and according to the form of N in *Penium margaritaceum* (Domozych, 2007). In addition, N can be stored extracellularly as EPS during deficiency (Boonchai et al., 2015), which suggests a reorientation of N metabolism. However, the diversity of metabolites produced by microalgal biofilms under N limitation remains, to our knowledge, little known.

Chlorophytes of the genus *Tetraselmis* are an industrially produced microalgae, mainly for biofuel (Montero et al., 2011) and aquaculture feed (Muller-Feuga et al., 2003), due to its lipid production profile (Huerlimann et al., 2010). Previous non-targeted metabolomic analyses also showed the presence of bioactive compounds of interest (Piampiano et al., 2021; Apostolopoulou et al., 2022). Notably, the species *T. suecica* shown potential for pharmaceutical and nutraceutical applications (Kellam et al., 1988; Kellam and Walker, 1989; Austin and Day, 1990; Austin et al., 1992; Lee et al., 2009; Custódio et al., 2014; Norzagaray-Valenzuela et al., 2017; Sansone et al., 2017; Guzmán et al., 2019; Haoujar et al., 2019; Hussein et al., 2020a, 2020b; Parra-Riofrío et al., 2020; Rentería-Mexía et al., 2022). Under N limitation, *T. suecica* tends to reduce its rate of sugar degradation (Lauritano et al., 2019) and accumulate carbohydrates, particularly starch (D’Souza and Kelly, 2000; Rodolfi et al., 2009; Kermanshahi-Pour et al., 2014), making it a potential candidate for bioethanol production (Lam and Lee, 2015; Lakatos et al., 2019). Conversely, transcriptomic shows little effect of N starvation on the transcripts involved in lipid metabolism, suggesting little changes in the lipid profile (Lauritano et al., 2019). However, these previous studies are only realised on planktonic culture. Recently, *T. suecica* has been used as a biofilm for biorefinery (Delran et al., 2024), showing increasing interest in using this species in this form. Nevertheless, it remains difficult to extrapolate the previous observations made on planktonic cultures of *T. suecica* under different N conditions onto its biofilms. Accordingly, it is necessary to study the influence of N status specifically on its monospecific phototrophic biofilm.

In this study, we examine the effect of N limitation on the metabolome of *Tetraselmis suecica* biofilm under laboratory conditions. In order to perform metabolomic analyses, a custom system was designed to satisfy three requirements: control of N levels while keeping other environmental parameters constant or "equal" (criterion I); accurate quantification of biological variables (criterion II); and rapid, contamination-free metabolite extraction (criterion III). Given the inherent challenges of biofilm systems, these criteria are difficult to meet compared to planktonic cultures. For criterion I, phototrophic biofilm thickness and its heterogeneity influence radiation field and photosynthesis. For criterion II, intrusive and partially destructive sampling is required to measure biofilm cell concentration, which may introduce bias into its metabolic response and therefore a non-compliance with criterion III. Moreover, estimating cell concentration from sub-sampling is impossible because of heterogeneity biofilm thickness. Here, we report metabolic and biochemical consequences of N limitation in *T. suecica* biofilms, including changes in adhesion behaviour. Additionally, we propose hypotheses linking N availability, metabolic profile changes, and adhesion dynamics, potentially mediated by nitric oxide (NO) signalling.

## 2 Materials and methods

### 2.1 Strains, medium and experimental conditions

*Tetraselmis suecica* strain AC254 was obtained from the algae collection of University of Caen (Algobank, France). The strain was maintained in batch cultures under continuous light intensity of 56 µmol·m^−2^·s^−1^ at 20 °C in a refrigerated incubator (IPP260, MEMMERT) and were routinely subcultured in cell culture flasks. The culture medium consisted of natural seawater taken from the subsurface in the bay of Villefranche-sur-Mer (France). After sea sourcing, the seawater was filtered through a 1.0 µm filter and then aged to remove residual nutrients before being filtered again through a 0.22 µm filter (Milligard filter cartridges, MILLIPORE) with a pump (Masterflex L/S model 7518–10), sterilised by autoclaving and enriched with modified f/2 medium without silicate (Table 1) (Guillard, 1975; Guillard and Ryther, 1962).

**Table 1:** Composition of the different media used.

Medium	[NO_3_^−^] (µM)	[PO_4_^3−^] (µM)	N/P	Other nutrients
f/2	882	36.3	24.1	as in f/2
2f’	3528	290.4	12.1	as in f/2
f/4′	441	36.3	12.1	as in f/2

Biofilms under N-replete (NR) and N-limited (NL) conditions were grown using two modified f/2 media with differing nitrate (NO_3_^−^) concentrations. Details of these media compositions and the N:P ratios are presented in Table 1 and further elaborated in Section 2.4.

We developed and optimized a dedicated biofilm culture system to reliably investigate the impact of N status on the metabolome of *T. suecica* biofilms while satisfying the criteria required for metabolomic analysis (i.e., environmental control, measurement accuracy, and contamination-free extraction). The culture system was designed to satisfy the following conditions: *(i)* no biological or chemical contamination, *(ii)* low-volume culture format, *(iii)* easy and rapid biofilm harvesting, *(iv)* sufficient replication (≥ 5 replicates per condition), *(v)* control and stability of all growth parameters (temperature, light, pH, nutrients except N), *(vi)* high reproducibility between replicates, and *(vii)* precise control of the N status of microalgae.

To this end, classical planktonic cultures were adapted to support development of contamination-free and significant microalgal biofilm in small volume. In order to obtain N status as only factor responsible for differences in metabolome response, others growth factors were selected to be non-limiting or maintained at equivalent levels between the two experimental conditions. Here, we assumed that the light conditions in harvested NR and NL biofilms were comparable if the biofilms had similar thickness. The Droop quota model (Droop, 1968) was used to define the initial experimental conditions for cell density and NO_3_^−^ concentration that, over an identical timeframe, would result in adequately concentrated cultures exhibiting distinct N statuses (for more details, see Supplementary Materials and Methods, and Supplementary Figures S1 to S5).

### 2.2 Cultivation and sampling conditions

For NL condition, ten autoclaved borosilicate glass Erlenmeyer flasks (Erlenmeyer ISO 1773, H: 135 mm, Ø: 34–79 mm, ref. FB33171, Fisherbrand™), equipped with a smooth PTFE magnetic bar (L: 25 mm, Ø: 6 mm, ref. 442–4505, VWR Collection) and a silicone stopper (Silicosen® T-type, H: 60 mm, Ø: 32 mm, ref. 8905,532, Schott Hirschmann) previously pierced in its centre with a cannula (microtube PFTE, ID: 1.32 mm, OD: 1.93 mm, Adtech Polymer Engineering) to provide humid filtered air and carbon (C) (Midisart® 2000, PTFE, 0.2 µm, 64 mm, 17,805——–UPN, Sartorius), were filled with 50 mL of f/4′ medium (Table 1) and inoculated with a *T. suecica* culture pre-acclimated in f/4′ medium to achieve an initial cell concentration of 10^8^ cell·L^−1^. Simultaneously, ten cultures in NR condition were prepared as previously with 50 mL of 2f' medium (Table 1) and inoculated with a *T. suecica* culture pre-acclimated in 2f' medium to reach an initial cell concentration of 10^7^ cell·L^−1^. Each condition also included two uninoculated Erlenmeyer flasks to serve as blanks for metabolomic analyses. The N:P ratio was set at 12:1 for both culture media. In total, twenty cultures and four blanks were incubated for 3 days in the incubation chamber (MLR-351, SANYO) at 25 °C (Sas et al., 2021), under continuous illumination of 540 µmol photons·m^−2^·s^−1^ (Montes-González et al., 2021) with agitation at 250 rpm using rotary magnetic stirrer (Colour squid white, ref. 0003,671,000, IKA®). Ten cultures and the four blanks were used for metabolomic analysis, while the remaining ten cultures were allocated for biochemical analysis (see *Supplementary Material and Methods*). Thus, since metabolite extraction requires complete biofilm destruction, five replicates per condition (NL and NR) were used for both metabolomic and biochemical measurements (Table 2).

**Table 2:** Experimental design. Crosses indicate the metabolomic and biochemical analyses performed on the various fractions of *Tetraselmis suecica* cultures under NR and NL conditions, as well as on the control samples.

		**Biofilm fractions**						**Blank**
		F-NA	F-L1	F-L2	F-L3	F-TL	F-DL	
**Metabolomic analyses**	Metabolome					**×**		**×**
**Biochemical analyses**	Salinity	**×**						**×**
	pH	**×**						**×**
	Particle count	**×**	**×**	**×**	**×**		**×**	
	Residual [NO_3_^−^]	**×**						
	Particulate N & C	**×**				**×**	**×**	
	Quantum yield (ɸ)	**×**				**×**	**×**	
	Pigments	**×**				**×**	**×**	

Once biofilm formed, the ten cultures for biochemical analysis were carefully removed from the incubation chamber, and magnetic stir bar removed using a magnetic rod to avoid contact with the culture medium. Biofilms were then fractionated into 6 components: non-adhered, planktonic cells (F-NA) were carefully recovered by decanting the supernatant; then, three sequential washes of the least adherent cell layers (F-L1, F-L2 and F-L3) using 50 mL of filtered seawater were performed. The top biofilm layer (F-TL) was obtained by mixing and then centrifuging (1500 rpm, 10 min; Centrifuge Sorvall™ ST 40R, ref. 75,004,525, Thermo Scientific) 10 mL of F-L1, F-L2, and F-L3 in a 50 mL conical tube. After removal of the supernatant, the pellet was resuspended in 10 mL of filtered seawater. Finally, deep biofilm layer (F-DL), composed of strongly adhered cells, was recovered by vigorous shaking with 50 mL of filtered seawater and magnetic stir bar for 5 min. These fractions are thus designated by their resistance to increasing agitation during their harvest, but not by a mechanical cutting with a fixed and previously determined thickness. All fractions were stored in 50 mL conical tubes (Nest® 50 mL, ref. 602,051, GROSSERON) for downstream analyses. The full biofilm (BIOF) was defined as the sum or average of the F-TL and F-DL fractions, depending on the analysis. Similarly, the entire culture (FULL) was defined as the sum or average of all previous fractions (F-NA, F-TL, and F-DL).

### 2.3 Biochemical analysis

#### 2.3.1 Cell density, cell diameter and biovolume

Depending on cell concentration, between 1 and 10 mL of F-NA, F-L1, F-L2, F-L3 and F-DL, fractions were individually diluted with filtered seawater to a final volume of 49 mL. Then, 1 mL of 10 % (v/v) HCl (hydrochloric acid, 37 %, extra pure, d = 1.18, SLR, ref. 10,000,180, Fisher Chemical) diluted in Milli-Q ultrapure water (SynergyPak® purification cartridge, ref. SYPK0SIA1, Merck) was added to disperse cell aggregates. Subsamples were analysed using an optical particle counter (OLS403, PAMAS Partikelmess- und Analysesysteme GmbH) which measures the distribution of cell sizes within a defined range (here, 2 **–** 20 µm). From these data, cell concentration, average cell diameter, and biovolume were calculated.

#### 2.3.2 Residual NO_3_^−^

A volume of 10 mL of F-NA fraction was filtered (Minisart® NML Sterile **–** 0.2 µm, SFCA, ref. 16,534———-K, Sartorius) and stored in cryotubes at −80 °C for subsequent analysis with an automated analyser (Technicon) (Tréguer, 1975).

#### 2.3.3 Particulate N and C

A volume of 5 mL of F-NA, F-TL and F-DL fractions was filtered (GF/F glass microfibre filters, Ø: 25 mm, circle, ref. 1825–025, Whatman®), then placed in haemolysis tubes pre-burned at 450 °C, sealed with aluminium foil and dried in an oven at 60 °C until analyses with a CHN analyser (Carlo Erba, model 1602).

#### 2.3.4 Pigments

A volume of 3 mL of F-NA, F-TL and F-DL fraction was filtered (GF/F glass microfibre filters, Ø: 25 mm, circle, ref. 1825–025, Whatman®), then flash-frozen in liquid N_2_ and stored at −80 °C until analysis by high-performance liquid chromatography (HPLC; HPLC Agilent Technologies, series 1200). The pigments quantified included: chlorophyll *a* (TChl_*a*), chlorophyll *b* (TChl_*b*) (detections at 667 nm), pheophytin *a* (Pheo_*a)*, neoxanthin (NeoX), violaxanthin (ViolaX), antheraxanthin (AntheraX), zeaxanthin (ZeaX), lutein, and unspecified carotenes (detections at 450 nm) (Ras et al., 2008).

#### 2.3.5 Quantum yield

Quantum yield of photosynthesis was estimated from 1 mL of F-NA, F-TL and F-DL fractions by measuring variable fluorescence using an AquaPen fluorometer (AquaPen-C AP110; PSI Photon Systems Instruments). In photosynthetic eukaryotes, the maximum theoretical quantum yield is typically around 0.7.

#### 2.3.6 Macromolecules

The relative proportions of carbohydrates, lipids, and proteins in the biofilms were estimated using attenuated total reflectance Fourier transform infrared spectroscopy (ATR-FTIR). Pellets obtained from 15 mL of centrifuged samples (1500 pm, 10 min) were washed twice with 10 mL of a 36 g·L^−1^ NaCl solution and stored at −80 °C until analysis. The remainder of the protocol follows the method described in Fanesi et al. (2019) (Fanesi et al., 2019). Briefly, pellets were resuspended in 5 to 10 µL of NaCl solution and 1.5 µL was transferred onto a ZeSe flat crystal at 45° and air-dried at room temperature for 20 min. Spectra were acquired using a spectrometer (PerkinElmer Spectrum-two, Waltham, MA) in reflection mode, scanning between 4000 and 400 cm^−1^. Spectra were determined using the elastic band algorithm and normalised to the amide I band. The ratios of the main macromolecular families – carbohydrates/proteins and lipids/proteins – were calculated from the maximum absorbance values in the following spectral regions: proteins (amide I; 1700 **–** 1630 cm^−1^), lipids (*C* = *O*; 1750 **–** 1700 cm^−1^) and carbohydrates (C—O-C, C—C and Si-O-Si; 1200 **–** 950 cm^−1^). Material analysed by ATR-FTIR included *T. suecica* cells and bacteria for the F-NA fraction, and a combination of microalgal cells, bacteria, and EPS matrix in the biofilm fractions. However, repeated washes and centrifugations during sample preparation likely altered the biological composition of the samples prior to analysis. Some loss of bacteria and EPS from the F-TL and F-DL fractions is expected.

#### 2.3.7 Salinity and pH

Salinity was estimated by depositing a drop of the F-NA fraction on a salinometer (Digital Salinity Refractometer MA887, Milwaukee) and pH was measured using a pH meter (pH-meter FP20 New FiveEasy, Mettler Toledo, pH electrode Bioblock Scientific, ref. 84,907, Fisherbrand™). For both measurements, NA fractions from biochemical and metabolomic samples were used.

### 2.4 Statistical analyses

Statistical analyses and figure generation were performed using RStudio (version 2024.04.2 + 764). The significance of differences in biochemical data between the two experimental conditions was assessed using *t*-tests, following verification of data normality with a Shapiro–Wilk test. One-way ANOVAs, followed by a Tukey post-hoc test, were used to compare fractions within the same experimental condition. Statistical significance was indicated as follows: p ≤ 0.05 (*), p ≤ 0.01 (**), and p ≤ 0.001 (***). Figures were manually edited using Inkscape software (version 1.3.2) to adjust colours, font sizes, and to add significance annotations or organize elements, without altering the underlying data or results.

### 2.5 Extraction of metabolites for metabolomic analysis

At the end of the incubation, the ten cultures and four blanks for metabolomic analysis were removed as previously with cultures intended for biochemical analyses (see *Supplementary Material and Methods*). Supernatants (F-NA fraction) were decanted as before and rapidly replaced with 40 mL of methanol [MeOH, liquid chromatography – mass spectrometry (LC-MS) grade, min. 99.9 %, LC-MS Ultra CHROMASOLV™, Honeywell Riedel-de Haën™), and then the Erlenmeyer flasks were placed in an ultrasonic bath for 20 min to accelerate the extraction of metabolites from the biofilm (condition *iii*). Performing the extraction in the same vessel in which the biofilm was grown greatly reduces the risk of contamination (condition *i*).

After transferring each obtained extracts to a conical tube (Centrifuge Sorvall™ ST 40R, ref. 75,004,525, Thermo Scientific), the remaining cell debris were removed by centrifugation (4000 rpm, 15 min), and the supernatant was transferred to another conical tube and stored at −80 °C. Unfortunately, one sample and one blank from the NL condition were lost during the process. The extracts were concentrated using a N_2_ evaporator (N-Evap) to estimate their mass, then transferred to a pre-weighed HPLC vial (2 mL, amber, ref. 5190–4034, Agilent Technologies) using a Pasteur pipette (230 mm, ref. 612–1702, VWR INTERNATIONAL) and finally evaporated to remove the residual solvent. The dry extracts were then weighed and stored at −80 °C under N_2_ until metabolomic analysis. Blanks were treated in the same way as the cultures.

### 2.6 Metabolomic analysis and LC-ESI(+)-MS and MS/MS data acquisition

Each dry extract was first solubilised in 1.5 mL MeOH (LC-MS grade, ref. 414,855, Carlo Erba), then filtered using a syringe (Plastic Two-piece Syringe S7515–3, 2 mL, ref. MB9202545TF-LAB, Thermo Scientific) fitted with a needle (22 *G* × 1 ¼"(0.7 × 32 mm), REGULAR BEVEL 11°, ref. AN*2232R1, AGANI NEEDLE) and a filter (Syringe Filter, 13 mm, 0.2 µm PTFE, Cat. #26,142, RESTEK) into an HPLC vial (Short Thread Vial 1.5 mL, 32 × 11.6 mm, ref. 11 09 0519, FISHERBAND) sealed with a screw cap fitted equipped with a septum (9 mm bleu UC Sil/PTFE, Cat. No. 11,787,567, FISHERBAND) to a final concentration of 4 mg mL^−1^ on average. The samples were analysed by liquid chromatography (Dionex Ultimate 3000 Rapid Separation, Thermo Fisher Scientific) coupled to a mass spectrometer (QToF Impact II, Bruker Daltonics, Mannheim) in positive mode [UHPLC-ESI(+)-QToF-HRMS/MS]. A quality control (QC) sample – containing 50 µL of each extract – was also injected using an insert (Cat. No. 11,762,418, FisherBrand) to 1/ stabilise the instrument, 2/ consider any MS drift, 3/ validate data treatment.

Chromatographic separation was carried out using a Kinetex® XB C18 column (150 × 2.1, 1.7 µm, Phenomenex) at 42 °C. Water (A) and acetonitrile (B), acidified with 0.1 % formic acid, were used as elution solvents. The flow rate of the mobile phase was set to 0.5 mL min^−1^. The elution program for chromatography was as follows: 10 % B for 2.5 min, a linear increase to 90 % B for 16.5 min, held at 90 % for 5.5 min, followed by a decrease to 10 % for 0.5 min, and then maintained for 3 min. In total, the analysis lasted 28 min. Mass spectrometry data were acquired from *m/z* 55 to 1200, in positive mode only. A QC sample was also injected in negative mode but did not exert any metabolite detection. The following MS parameters were used for QToF: end plate offset at 500 V, nebulizer gas pressure (N_2_) at 3.5 bars, dry gas flow rate (N_2_) at 12.0 L·min^−1^, drying temperature at 200 °C, MS^1^ acquisition frequency at 2 Hz, and capillary voltage at 4500 V. MS/MS fragmentation spectra were obtained automatically for the 3 most abundant precursor ions. The MS/MS data acquisition mode was set with a scan frequency of 8 Hz and a collision energy of 20 **–** 40.0 eV (50 % time at each collision energy, stepping mode).

### 2.7 Processing and analysis of metabolic data

Once the UHPLC-ESI(+)-QToF-MS/MS data have been acquired, the raw data cannot be used directly due to the very large number of detected ions (i.e. ionised metabolites generated during analysis). A dedicated data processing and statistical analysis workflow, specific to metabolomic datasets, was required to list, select, and characterise ions whose intensity varied according to N status. This workflow relied on multiple specialized software tools and step-by-step by the user. For this purpose, raw LC-MS data were calibrated using Bruker DataAnalysis software (version 5.0) and converted to .mzXML format using the MSConvert protocol (version 3.0.23345-eb399e3) (Chambers et al., 2012). The offset obtained from the precursor ions during conversion was then corrected using the script available at https://github.com/elnurgar/mzxml-precursor-corrector (version 0.5; see (Breaud et al., 2022; elnurgar, 2024) for details). The resulting .mzXML data were pre-processed using MZmine3 (version 3.9.0). After blank subtraction, the dataset was exported in .csv and .mgf formats. Manual filtering was applied to retain only those ions with a coefficient of variation (CV) <25 % in QC samples before performing statistical analyses.

Three multivariate statistical methods were applied: *(i)* principal component analysis (PCA), *(ii)* partial least squares discriminant analysis (PLS-DA), and *(iii)* heatmap. Multivariate statistical analyses were performed using MetaboAnalyst web platform (version 6.0; https://www.metaboanalyst.ca/) (Pang et al., 2024) to identify potential contaminants and the most discriminating ions (Variables Importance in Projection, VIPs) between NL and NR conditions. Contaminants were identified based on PCA results and manually excluded. VIPs were identified *via* PLS-DA and heatmap analyses with LOOCV for cross validation method, then further characterised using databases (CAS Scifinder, LipidMaps, SIRIUS, GNPS) (Dührkop et al., 2019, 2015) and MetFrag *in silico* tool. A VIP score threshold of 1.3 was chosen to select only highly discriminating ions. For each VIPs, the intensity differences between the two experimental conditions were confirmed using the univariate Student's *t*-test, with False Discovery Rate (FDR) correction using Benjamini-Hochberg method. Finally, a molecular network was generated by importing the data into GNPS (https://gnps.ucsd.edu/ProteoSAFe/static/gnps-splash.jsp) and visualised using Cytoscape software (version 3.10.1). Data and molecular network are freely available and deposited on Zenodo (10.5281/zenodo.15834877). The entire workflow of processing and filtering metabolomic data is described in the *Supplementary table* (tabs 0 to 3) and summarized in *Supplementary figure S6*.

## 3 Results and discussion

To accurately analyse the metabolic response of *T. suecica* biofilms to changes in N status, three conditions had to be fulfilled. First, the metabolomic analysis had to be conducted on cultures clearly exhibiting different N statuses (NL and NR). Second, other growth factors that could influence the metabolic response – light, temperature, salinity, pH, and nutrients other than N – had to be either non-limiting or identical between the two experimental conditions.

### 3.1 Validation of different N statuses

As expected, residual NO_3_^−^ concentrations at the end of the experiment were above 100 µM in NR cultures and undetectable in NL cultures (data not shown).

**Fig. 1:**
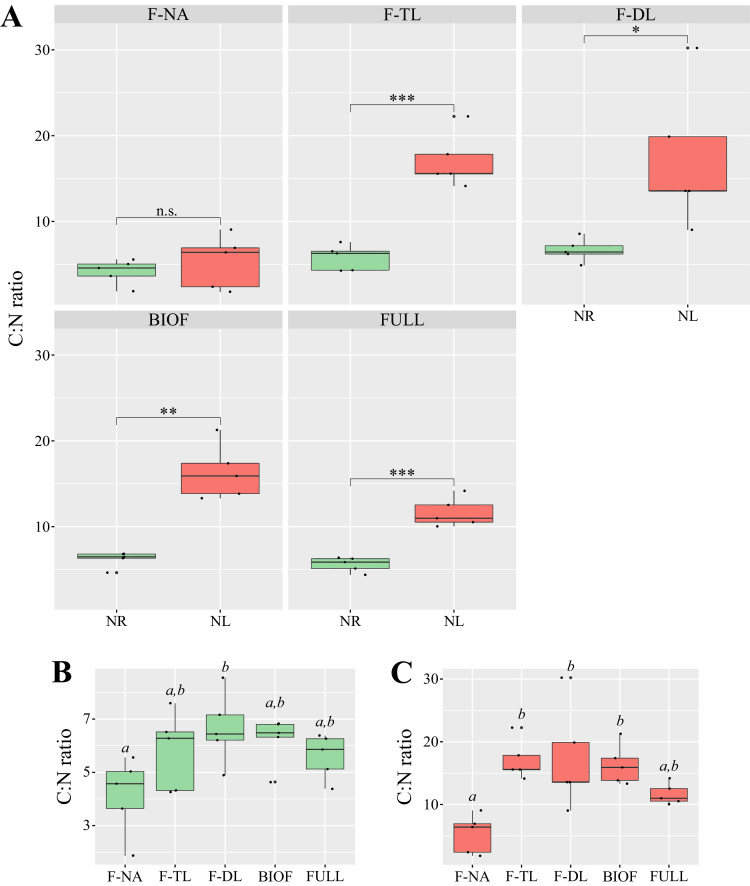
Variation in the C:N ratio across *Tetraselmis suecica* biofilms. C:N ratios in nitrogen-replete (NR; green) and nitrogen-limited (NL; red) biofilms and culture fractions (A). n.s.: not significant; *: p-value < 0.05; **: p-value < 0.01; ***: p-value < 0.001. C:N ratios in the individual fractions of NR (B) and NL (C) cultures. Fractions: non-adherent cells (F-NA), top layer (F-TL), deep layer (F-DL), biofilm (BIOF = F-TL + F-DL), and all fractions combined (FULL = F-NA + F-TL + F-DL). Statistical differences are indicated by different italic letters above box plots.

The C:N ratio measured in NR and NL biofilms was 6.2 ± 1.4 and 17.2 ± 5.9, respectively (Fig. 1**A**), indicating that NR biofilms contained more than twice as much N as NL biofilms (*p* value < 0.01; *t*-test), although quota C may also vary to a lesser extent. However, it should be noted that these ratios represent the average C:N ratio of all particles retained on the filter – including microalgal cells, bacteria, EPS, and other matrix components – some of which (e.g., EPS) are C-rich. This average was weighted by the relative abundance of the F-TL and F-DL fractions. Nevertheless, if the biofilm is considered a unified living structure, the C:N ratios remained clearly distinct between the two experimental conditions across all biofilm fractions (Figs. 1**A, B, C**). These results confirm that our protocol effectively yielded biofilms with contrasting N statuses (condition *vii*; see Sections 2.2 and *Supplementary Material and Methods*). However, our data do not allow us to determine which process(es) most strongly contributed to this divergence in C:N ratios – whether it was a modification of the N status of microalgal cells or a change in EPS production. The C:N ratios measured in F-NA fractions of NR and NL cultures averaged 4.1 ± 1.4 and 5.3 ± 3.1, respectively (Fig. 1**A**). The stronger divergence observed in the F-TL and F-DL fractions compared to the F-NA fraction (Fig. 1**B and C**) is more difficult to attribute solely to changes in microalgal N status, given the presence of EPS in the biofilm. However, the higher C:N ratio in the deepest layers (F-DL compared to F-TL) may reflect a gradient in NO₃⁻ availability, decreasing with biofilm depth. The presence of such a gradient cannot, nevertheless, be confirmed due to the limits of our biofilm harvesting and fractionation protocol. Although offering conclusive overall results (BIOF), our protocol only allows us to subdivide our biofilms into two cellular layers (F-DL and F-TL) depending on the adhesion of the cells to each other and to the substrate. Thus, our results present a significant simplification of the internal organization of the biofilm of *T. suecica*. In order to estimate this potential gradient of NO_3_^−^ availability, other quantitative and qualitative measurements of nutrients on biofilms of different thicknesses and stratified by layers of fixed thickness are necessary.

**Fig. 2:**
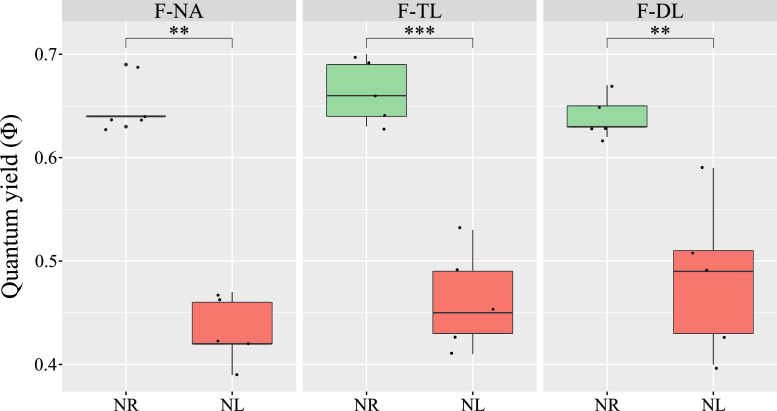
**Photosynthetic efficiency across the biofilm under nitrogen limitation.** Quantum yield (ϕ) measured in fractions from nitrogen-replete (NR; green) and nitrogen-limited (NL; red) cultures. **: p-value < 0.01; ***: p-value < 0.001. Fractions: non-adherent cells (F-NA), top layer (F-TL), and deep layer (F-DL).

The quantum yield of photosynthesis measured at the end of the experiment ranged from 0.6 to 0.7 across the different fractions of NR biofilms (Fig. 2). These differences between fractions were not statistically significant, suggesting that the slight variations in C:N ratios observed among the same fractions (Fig. 1**A**) did not affect their photosynthetic efficiency, which remained close to the theoretical optimum of 0.7. In contrast, quantum yield values measured in NL fractions were significantly lower (p < 0.05; *t*-test), with most values falling below 0.5, indicating impaired photosynthetic performance under NL conditions.

**Fig. 3:**
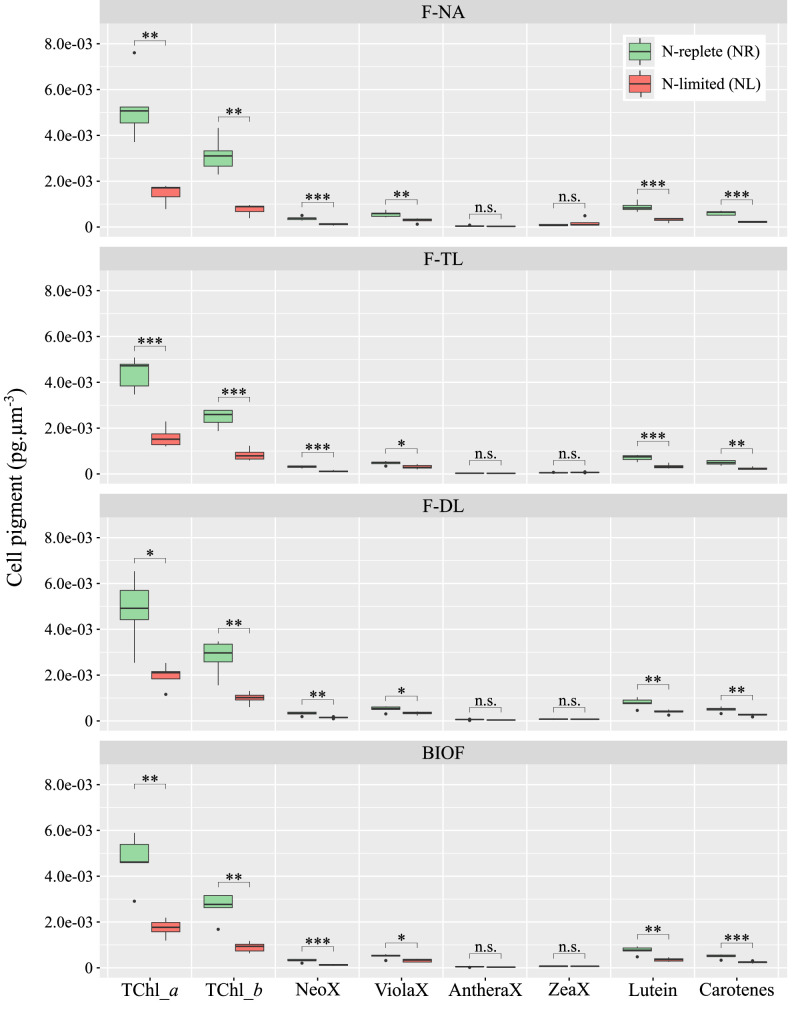
Profiles of photosynthetic and secondary pigments under nitrogen limitation. Pigments measured in nitrogen-replete (NR; green) and nitrogen-limited (NL; red) biofilms and culture fractions, normalized by biovolume. Pigments: total chlorophyll *a* (TChl_*a*), total chlorophyll *b* (TChl_*b*), Neoxanthin (NeoX), Violaxanthin (VioX), Antheraxanthin (AntheraX), Zeaxanthin (ZeaX), Lutein, and Carotenes. n.s.: no significant difference; **: p-value < 0.01; ***: p-value < 0.001.

The cellular content of most pigments measured at the end of the experiment was significantly lower in NL than in NR biofilms across all fractions (Fig. 3). This reduction – approximately 18 % for both TChl_*a* and TChl_*b* – was consistent across the different fractions (TChl_*a: p* values < 0.01; TChl_*b: p* values < 0.05; *t*-test). It is also worth noting that the cellular contents of TChl_*a* and TChl_*b* within NL and NR cultures did not differ significantly between the fractions.

In conclusion, the data on residual NO_3_^−^, C:N ratios, quantum yields and pigments contents are all consistent with distinct N statuses between the two experimental conditions.

### 3.2 Validation of the conformity of other growth factors

The arrangement of the Erlenmeyer flasks within the incubation chamber ensured virtually identical temperatures across all cultures (see *Supplementary Material and Methods*). We consider that the slight variations had only a minimal impact on the metabolomic analysis.

Salinity ranged from 38 to 41 g·L^−1^, with no significant differences between NL and NR conditions (*Supplementary figure S7*). These minor variations in salinity likely resulted from slight differences in evaporation (and consequently bubbling) between cultures and were accounted for in the final calculation of cell concentrations in the F-NA fractions.

**Fig. 4:**
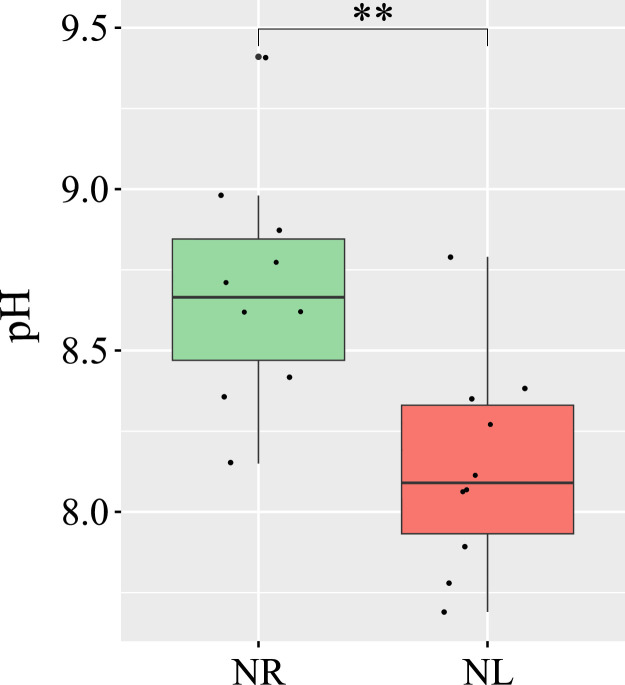
Final pH in the supernatant of nitrogen-replete and nitrogen-limited cultures. pH measured in the F-NA fraction of nitrogen-replete (NR; green) and nitrogen-limited (NL; red) cultures at the end of the experiment. **: p-value < 0.01.

At the end of the experiment, pH remained below 9.0 in most NL and NR cultures (Fig. 4), a level at which CO_2_ availability is not considered limiting for photosynthesis (Zeebe and Wolf-Gladrow, 2001). However, pH was significantly higher in NR cultures, consistent with greater CO₂ uptake due to higher photosynthetic activity. While this difference likely did not affect growth, we cannot exclude the possibility that it influenced the physiology of *T. suecica* biofilms and, consequently, their metabolomic profiles.

**Fig. 5:**
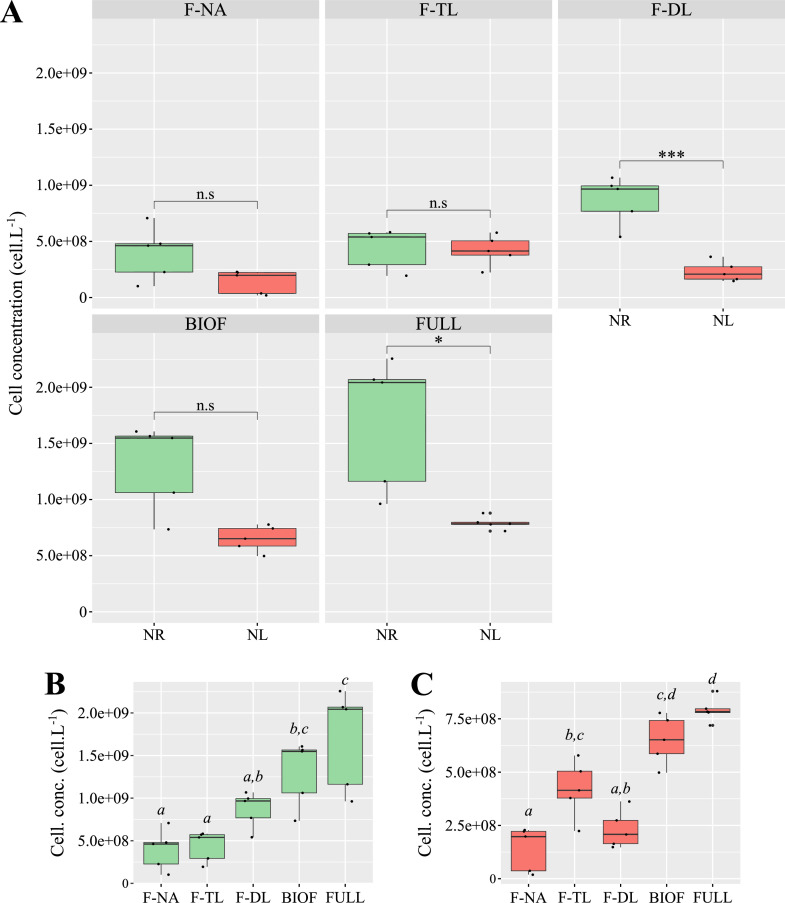
**Cell concentration across *Tetraselmis suecica* biofilms.** Cell concentration measured in nitrogen-replete (NR; green) and nitrogen-limited (NL; red) biofilms and culture fractions (A). n.s.: no significant difference; *: p-value < 0.05; and ***: p-value < 0.001. Cell concentration of NR (B) and NL (C) cultures fractions. Fractions: non-adherent cells (F-NA), top layer (F-TL), deep layer (F-DL), biofilm (BIOF = *F*-TL + *F*-DL), and all fractions combined (FULL = F-NA + F-TL + F-DL). Differences are significant when italic letters above box plots are different.

**Fig. 6:**
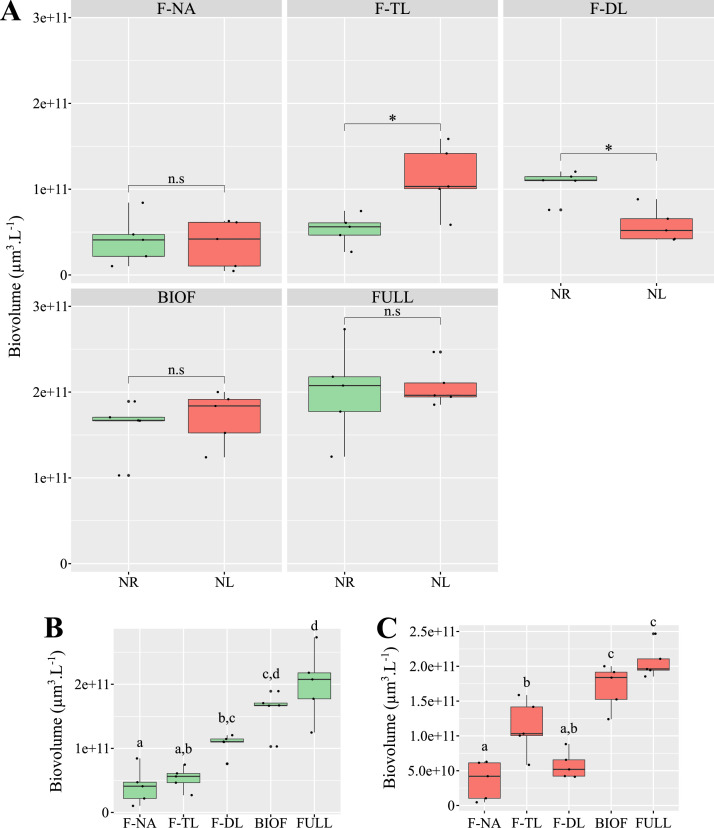
Biovolume across *Tetraselmis suecica* biofilms. Biovolume measured in nitrogen-replete (NR; green) and nitrogen-limited (NL; red) biofilms and culture fractions (A). n.s.: no significant difference; and *: p-value < 0.05. Biovolume of NR (B) and NL (C) cultures fractions. Fractions: non-adherent cells (F-NA), top layer (F-TL), deep layer (F-DL), biofilm (BIOF = F-TL + F-DL), and all fractions combined (FULL = F-NA + *F*-TL + *F*-DL). Differences are significant when italic letters above box plots are different.

Regarding light, we hypothesized that similar cell concentrations at the time of sampling would imply comparable light gradient within the biofilms under both experimental conditions, assuming as a first approximation that biofilm thickness correlates with cell concentration. Cell concentration was significantly two-fold higher in NR biofilms (1.30 ± 0.39 × 10^9^ cell·L^−1^) than in NL biofilms (6.51 ± 1.14 × 10^8^ cell·L^−1^) on day 3 (Fig. 5). In contrast, biovolumes did not differ significantly between NR (1.59 ± 0.33 × 10^11^ µm^3^·L^−1^) and NL biofilms (1.70 ± 0.32 × 10^11^ µm^3^·L^−1^) (Fig. 6), indicating that average cell diameter was significantly greater in NL biofilms (*Supplementary figure S8*). This increase in cell diameter was observed across all fractions. According to the Droop model, similar algal cell densities were predicted in NL and NR biofilms after 3 days of incubation (*Supplementary figure S5*). However, it is worth noting that the cell concentration in the NR cultures was still increasing rapidly at day 3, as cells were in the exponential phase of growth. In an unsynchronized microalgal cell population like ours – due to continuous illumination – cells are at various stages of the cell cycle. During the transition from mother cell to daughter cell, somatic growth increases cell size until division occurs. Thus, in NR cultures, the average cell diameter reflects a mix of mother and daughter cells. Under N limitation, however, an increasing number of cells are unable to divide and become arrested in the pre-mitotic phase of the cell cycle, where they approach the size of a mother cell. As a result, the average diameter of the NL population is larger than that of a non-limited population. An increase in cell diameter under nutrient limitation has frequently been reported in microalgae (Mocquet et al., 2013; Yap et al., 2016). Therefore, our N limitation protocol not only affects C and N metabolism differently under NL and NR conditions, but also alters the distribution of cell cycle stages within the populations.

Using biovolume rather than of cell concentration is therefore, in our view, a more appropriate proxy for estimating biofilm thickness, and consequently the potential differences in light penetration, between NL and NR conditions. Under this assumption, the biofilms in both conditions can be considered to have comparable thicknesses and to be similarly affected by light attenuation. However, it should be noted that this remains a partial estimate of biofilm thickness, as no measurement of the EPS matrix content was performed in this study. Indeed, in some bacterial biofilms, the EPS matrix can represent up to 90 % of the dry biomass (Flemming and Wingender, 2010).

Additionally, NL biofilms exhibited an increased biovolume in the F-TL fraction at the expense of the F-DL fraction, suggesting reduced cell adhesion within the biofilm structure (Fig. 6). Biofilm detachment may represent a physiological response to nutrient depletion and subsequent deficiency (Hunt et al., 2004). However, no significant difference in the biovolume of the F-NA fraction was observed between the two conditions (Fig. 6), indicating that the loss of adhesion did not result in measurable cell detachment. This may reflect an early-stage nutritional stress, occurring too soon to trigger active cell release. The timing of sampling may therefore have preceded the onset of detachment mechanisms typically associated with more prolonged or severe N limitation. As previously, additional imaging and compositional data of EPS involved in adhesion on stratified biofilms are needed to confirm our interpretations.

No phosphate (PO_4_^3−^) measurements were performed. However, as previously mentioned (see Section 2.2), the culture media were formulated with an excess of PO_4_^3−^ relative to NO_3_^−^, following to the Redfield ratio, in order to prevent phosphorus from becoming a limiting factor. It has been shown that N-deprived *Chlamydomonas reinhardtii* cells simultaneously reduce their PO_4_^3−^ uptake (Kamalanathan et al., 2016). We therefore assume that the N:P ratio was not significantly different between the NL and NR conditions at the time of sampling.

Taken together, these results suggest that the necessary conditions for conducting a robust metabolomic study were almost entirely fulfilled.

### 3.3 Macromolecule composition in *T. suecica* biofilms is affected by N limitation

**Fig. 7:**
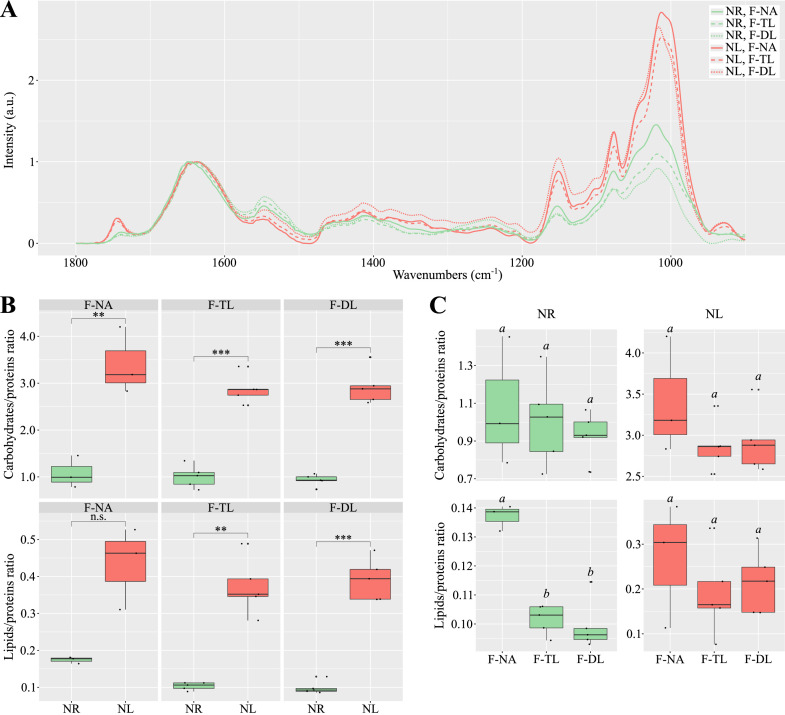
**Macromolecular shifts in response to nitrogen status.** Relative carbohydrate, lipid and protein compositions in nitrogen-replete (NR; green) and nitrogen-limited (NL; red) culture fractions estimated by Fourier transform infrared spectroscopy (ATR-FTIR). A) Spectra normalized to the maximum of the amide I band (1700–1600 cm^−1^). B) Carbohydrate/protein and lipid/protein ratios for NR (green) and NL (red) culture fractions. n.s.: no significant differences; **: p-value < 0.01; and ***: p-value < 0.001. C) Carbohydrate/protein and lipid/protein ratios for NR (green) and NL (red) culture fractions. Differences are significant when italic letters above box plots are different.

After data normalisation, the amide I band in the FTIR spectra exhibits a shift towards 1640 cm^−1^ under NL condition, and towards 1650 cm^−1^ under NR condition (Fig. 7**A**). Overall, the spectra share a similar profile: a band around 1650–1640 cm^−1^ (amide I), another near 1545 cm^−1^ (amide II), a broad undifferentiated region between 1480 and 1190 cm^−1^, and three peaks around 1150, 1075 and 1015 cm^−1^ (carbohydrates).

The peaks at 1745 cm^−1^, likely corresponding to lipids and fatty acids (FAs), are weak under NR condition but clearly visible in all fractions under NL condition. No major differences in relative intensity at this range are observed between fractions under both conditions. However, the peaks in the amide II band appears slightly less intense in NL condition, regardless of the fraction. For this spectral region, intensity differences between fractions in both conditions are minor and follow the order: F-DL > *F*-TL > *F*-NA. Putative carbohydrate-associated peaks are consistently less intense under NR conditions than under NL conditions, regardless of the specific peak or fraction. Under NR conditions, the F-NA fraction displays higher intensity than the biofilm fractions. No clear differences are observed between these two biofilm fractions with the exception of the peak at ∼1015 cm^−1^, where the intensity of the F-DL fraction is lower than that of F-TL. Under NL conditions, relative intensity patterns differ, although the top biofilm layer consistently shows the weakest signal. Overall, the FTIR spectra follow a similar profile to other N-deficient microalgae (Wagner et al., 2019).

Potential relative macromolecular ratios were calculated (Fig. 7**B**). Overall, if we consider the peaks cited as associated only with macromolecules, NL biofilms contain significantly higher levels of carbohydrates and lipids, but lower levels of proteins compared to NR biofilms (*p* values < 0.01; *t*-test). Only the F-NA fraction shows a notable but non-significant difference for lipids/proteins ratio (*p* value = 0.055; *t*-test) between both treatments. Our results may suggest that N limitation increased the lipids/proteins and carbohydrates/proteins ratios in *T. suecica* biofilms, which could reflect either a reduction in proteins, an increase in lipids and carbohydrates, or both.

Protein reduction and lipid accumulation are common responses to N limitation in microalgae (Kamalanathan et al., 2016; Tarazona Delgado et al., 2021; Yaakob et al., 2021), although these responses can vary among species. Lim et al. (2017) (Lim et al., 2017) reported that N-limited *Tetraselmis* sp. M8 initially exhibited a decrease in lipid consumption, followed by active production and intracellular accumulation of saturated (SFAs) and monounsaturated (MUFAs) FAs. These results corroborate those of Adarme-Vega et al. (2014) (Adarme-Vega et al., 2014), who observed that the FAs biosynthesis pathways in this species are positively regulated under combined N and P limitation.

However, *T. suecica* exhibits a distinct physiological response. Similar to the chlorophyte *Isochrysis zhangjiangensis* (Feng et al., 2011), *T. suecica* tends to favour either lipids or carbohydrates production depending on whether N is singly or co-limiting. Indeed, D'Souza & Kelly (2000) (D’Souza and Kelly, 2000) reported that N-deficient conditions led to a threefold increase in carbohydrate content in strain CS-187, while protein and lipid content decrease slightly – a trend also observed by Lourenço et al. (1997) (Lourenço et al., 1997) in *T. gracilis* C1. Similarly, in *T. suecica* F&M-M33, co-limitation by N and P had no effect on lipid content, but resulted in a >50 % increase in carbohydrate content (Rodolfi et al., 2009; Bondioli et al., 2012). In *T. suecica* CCMP906, these physiological responses are also mirrored at the transcriptional level, with decreased expression of genes involved in amino acid synthesis and carbohydrate degradation under N limitation (Lauritano et al., 2019). Conversely, transcripts involved in lipid biosynthesis remain unaffected by N availability (Lauritano et al., 2019). Starch is the primary storage form of carbohydrates in *T. suecica* (Kermanshahi-Pour et al., 2014), a pattern also observed in *T. subcordiformis* (Yao et al., 2012, 2013; Meng et al., 2014; Jiang et al., 2017), which assumes that the observed increase in carbohydrates is mainly composed of this polysaccharide. An increase in lipid content exclusively under N-rich medium was also observed in *T. marina* CTM 20,015 (Dahmen-Ben Moussa et al., 2017) and *Tetraselmis* sp. KCTC 12236BP (Kim et al., 2016).

Moreover, the increase in lipid content in *T. suecica* appears to require a relatively prolonged period of N deficiency (Rodolfi et al., 2009), which was not achieved in our experiment. In contrast, starch biosynthesis is known to begin rapidly following the onset of N limitation, before gradually declining over time (Yao et al., 2012). All these previous observations suggest a hypothetical macroscopic reduction in protein synthesis and concurrent accumulation of carbohydrates in the form of starch in our NL biofilms of *T. suecica* AC254. Therefore, the observed relative increase in lipids compared to proteins would most likely reflects a decrease in protein levels, as N limitation restricts amino acid biosynthesis. Nevertheless, interpretations of our ATR-FTIR measurements should be approached with caution, given the complex composition of *T. suecica* biofilms – including microalgal cells, bacteria, and EPS matrix – and the unknown proportion of each component retained on the filter and then after successive washes. Moreover, the EPS matrix of biofilms contains high levels of polysaccharides, enzymes, and lipids, with relative proportions that may differ significantly from those in suspension cultures (Flemming and Wingender, 2010; Bharti et al., 2017). As the current literature primarily focuses on planktonic systems, direct comparisons with our biofilm data remain limited.

Fig. 7**C** indicates no significant difference in macromolecular ratios between fractions within each experimental condition, except for a markedly higher putative lipid abundance in the supernatant compared to the biofilm (*p* value < 0.0001; one-way ANOVA). Finally, the assumed carbohydrate-to-protein ratio in NL cultures appears to increase with biofilm depth. These differences in assumed lipid-to-protein ratios between suspended and biofilm-associated cells under NR condition suggest that adhered cells may be less lipid-rich, more protein-rich, or both. However, although our results seem *a priori* consistent with those obtained on planktonic cultures, further investigations are needed to improve our understanding of the composition and structure of the EPS matrix in monospecific microalgal biofilms.

Monitoring the N status of *T. suecica* biofilms therefore appears to be a promising strategy for steering macromolecular production toward specific compound families, with potential applications in the biofuel industry. In particular, its hypothetical strong production of starch could have a great interest for the production of bioethanol, thermoplastic biopolymers, or even starch-based feedstocks (Marques et al., 2018). The EPS matrix can also be considered as an external digestive system by retaining enzymes excreted by the cells, thereby supporting various functions (Flemming and Wingender, 2010). This functional role may partially explain the observed differences in protein content between cells in the supernatant and those embedded within the biofilm, but this hypothesis requires further investigation.

### 3.4 Metabolic and lipid profiles change in N-limited *T. suecica* biofilms

**Fig. 8:**
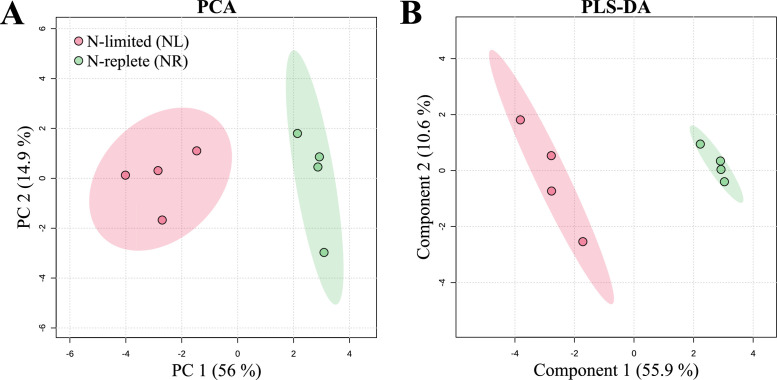
**Multivariate analysis of *Tetraselmis suecica* biofilm metabolomes.** Principal Component Analysis (A) and Partial Least Squares Discriminant Analysis (B) of UHPLC-ESI(+)-QToF-MS data from MeOH extracts of nitrogen-replete (NR; green) and nitrogen-limited (NL; red) biofilms. Analyses and plots were generated using MetaboAnalyst (v6.0).

LC-ESI(+)-MS metabolomic analysis initially identified 7 627 m*/z* features in the raw LC-MS data, of which only 227 remained after three filtration steps and blank subtraction (see *Supplementary table*). In the initial PCA projection, QCs sample were positioned between NL and NR conditions on the second axis, but clearly shifted altogether along the first axis. This shift enabled the identification and manual removal of numerous contaminants in the QCs sample (see *Supplementary table* for further details). After this cleaning, the PCA showed well positioned QCs between groups NR and NL, as well as a clear distinction between these two conditions (PC1 = 38.7 %, PC2 = 27.4 %; p-value < 0.05, based on 999 permutations; Permanova test) (see *Supplementary table*). Additionally, one replicates of the NR condition that displayed an intermediate metabolic profile between the two conditions in the PCA. In order to better characterize the metabolome differences in *T. suecica* biofilms during N limitation, this intermediate profile replicate was excluded to enhance the identification of the most discriminating ions (see *Supplementary table*). After cleaning the dataset, only 120 m*/z* features remained and the PCA distinctly separated NL and NR sample groups, with a robust projection quality (p-value < 0.05, based on 999 permutations; Permanova test). The first two principal components accounted for 70.9 % of the total variance (Fig. 8**A**), with the separation primarily occurring along PC1, which alone explains 56 % of the variance. These results confirm that N limitation had a significant effect on the metabolome of *T. suecica* in biofilm.

Consistent with the PCA results, PLS-DA projection logically shows a distinction between NL and NR samples, with satisfactory representativeness (the first two axes account for 66.5 % of the total variance) (Fig. 8**B**) and high predictive accuracy and relevance (R^2^ > 0.99, Q^2^ = 0.90, cross validation) but no significance (*p* value = 1; permutation test), which suggests that this separation is due to randomness and that an overfitting is present due to the multiple previous filtrates (see *Supplementary table*). However, PLS-DA performed before filtrations also showed no significant difference, which could be caused by the very strong biological proximity of our samples in our experiment. A total of 36 m*/z* features with higher discriminating metabolites (VIP score > 1.3) were selected: 26 features were overexpressed in the NR condition, while 10 were overexpressed in the NL condition (*Supplementary figure S9*). Among the 36 features of interest, several are identical but either represent different adducts or correspond to *In-Source* fragments (ISF). After accounting for these redundancies, a total of 24 unique metabolites were finally annotated, of which 17 were overexpressed in the NR condition and 7 in the NL condition. Due to its non-significance, our PLS-DA cannot confirm that there is a marked profile of expression between these metabolites. Thus, our two conditions were compared using univariate tests on each identified VIPs to verify their intensity differences, which helped us clarify the observed overfitting. Except for two VIPs (4332 and 6469), all annotated metabolites showed significantly different intensities between the two conditions (p value < 0.05; Student’s *t*-test) (see Table 3 and *Supplementary table*), supporting the previous profile obtained via multivariate analyses.

**Table 3:** Impact of nitrogen limitation on the *Tetraselmis suecica* biofilm metabolome. Fold changes for 13 annotated metabolites among the 21 most discriminant features listed *via* PLS-DA (based on VIP scores), comparing nitrogen-replete (NR) and nitrogen-limited (NL) conditions. Metabolites downregulated under NL conditions are shown in blue; upregulated ones are shown in red (for details on metabolite annotation, see supplementary table). *: p-value < 0.05; **: p-value < 0.01; and ***: p-value < 0.001.

**Fig. 9:**
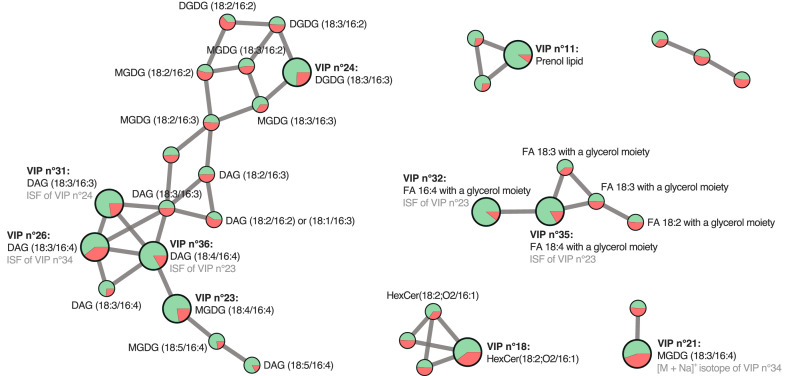
**Molecular network of LC-MS/MS metabolomic data highlighting clusters of interest.** Metabolites are represented as nodes, clustered based on spectral similarity. VIPs identified via PLS-DA are indicated by enlarged circles. Node colours reflect relative abundances under nitrogen-replete (green; NR) and nitrogen-limited (red; NL) conditions. Abbreviations: monogalactosyldiacylglycerol (MGDG); digalactosyldiacylglycerols (DGDG); diacylglycerol (DAG); fatty acid (FA); glycosphingolipids (HexCer); and *In-Source* fragments (ISF).

From the MS/MS data, five glycosyldiacylglycerols and two glycosphingolipids (HexCer) were partially characterised (Table 3). The glycosyldiacylglycerols include three monogalactosyldiacylglycerols (MGDGs) and two digalactosyldiacylglycerols (DGDGs). The positions of the double bonds in the C chains of FAs or sphingosines groups of these molecules could not be determined. All of these metabolites are overexpressed in the NR condition (see *Supplementary table* for further details). None of the metabolites overexpressed in the NL condition could be identified, although some of them could be sphingolipids according to their molecular formulae and MS/MS spectra. Molecular networking analysis additionally revealed five more MGDGs, two other DGDGs, one other HexCer, which were not among the VIPs, as well as 8 diacylglycerols (DAGs) and 5 FAs, which are likely ISFs of MGDGs or DGDGs, whether they are VIPs or not (Fig. 9). Notably, among these compounds, only FA 18:3 bound to a glycerol moiety (ion 5782) and MGDG (18:3/16:3) (ion 6014) exhibited a VIP score > 1. However, the molecular networks could not be fully elucidated, and several metabolites upregulated in the NR condition remain unknown.

Our metabolomic data reveal differences in the lipid profiles of *T. suecica* biofilms. D'Souza & Kelly (2000) (D’Souza and Kelly, 2000) similarly reported that macronutrient limitation alters the lipid composition of *T. suecica* F&M-M33, notably increasing the proportion of SFAs and MUFAs compared to polyunsaturated fatty acids (PUFAs). Among these FAs, palmitic acid (16:0), oleic acid (18:1 n-9), linoleic acid (18:2 n-6), linolenic acid (18:3 n-3) and eicosapentaenoic acid (20:5 n-3) are highly represented (D’Souza and Kelly, 2000; Bondioli et al., 2012), with palmitic acid dominating in N-limited cells (Go et al., 2012). The biosynthesis of glycosyldiacylglycerols and glycosphingolipids could be redirected toward the production of other lipids, such as FAs. In parallel, these glycolipids could undergo degradation as N limitation progresses, with their carbohydrate groups acting as polar heads potentially contributing to starch synthesis. However, the change in lipid composition under N limitation does not necessarily correspond to a straightforward conversion of polar lipids into neutral lipids. In fact, the content of certain FAs such as 16:0, 18:1 n-7, 18:2 n-6 and 20:4 n-6 in *T. suecica* F&M-M33 increases under N-limited conditions, whereas others, including 16:1 n-9, 16:4 n-3, 18:3 n-3 and 18:4 n-3 FAs, decrease. Others, such as 20:5 n-3, appear to remain unaffected (D’Souza and Kelly, 2000). In other microalgae, the lack of NO_3_^−^ increases the levels of MGDGs and DGDGs (Wang et al., 2016), although this trend is not observable in all N-deficient microalgal species (Simionato et al., 2013). Conversely, although the overall carbohydrate content rises, primarily as starch, the monosaccharide composition appears to remain stable. This composition is mainly comprised of 3-deoxy-*d*-manno‑oct-2-ulosonic acid (also known as Kdo), followed by 3-deoxy-lyxo-2-heptulosaric acid (Dha) and galacturonic acid, and finally galactose (Kermanshahi-Pour et al., 2014).

MGDGs and DGDGs are key structural glycoglycerolipids of thylakoid membranes (Goss and Latowski, 2020). MGDGs are particularly involved in the regulation of the xanthophyll cycle, which plays a central role in non-photochemical quenching (NPQ) in plants and microalgae (Goss and Latowski, 2020; Ruban, 2016). Under high light intensity and low pH, MGDGs can form non-bilayer lipid phases that facilitate the conversion of VioX to AntheraX and ZeaX, two pigments essential for NPQ photoprotection. In NL conditions, the downregulation of specific MGDGs (Table 3), along with a decrease in ViolaX content (Fig. 3), suggests an impaired photoprotective capacity in *T. suecica* biofilms. However, the lack of a significant rise in AntheraX and ZeaX concentrations indicates a stable rate of photodegradation, despite the presumed decrease in photoprotective capacity. We propose that the reduced light penetration in the *T. suecica* biofilm may partially compensate for the diminished NPQ capacity.

Our results provide clear evidence that N deficiency significantly impacts the metabolome – particularly the lipidome – of *T. suecica* biofilms. However, the identification of discriminating metabolites remains incomplete, especially under the NL condition, where no metabolites could be confidently identified. Moreover, among the characterised compounds, only MGDG(18:3/16:4) and MGDG(18:4/16:4) have been reported in the literature to exhibit known biological activity (Banskota et al., 2013). The absence of established biological functions for most of the identified metabolites considerably limits our ability to interpret their ecological or physiological relevance. Further analyses are therefore necessary to more accurately identify, characterise, and elucidate the roles of the lipids and other metabolites involved.

### 3.5 A hypothetical role of NO in impairing *T. suecica* biofilm adhesion

We present below an exploratory, inference-based hypothesis proposing a mechanism by which NO production may regulate the adhesion of monospecific microalgal biofilms.

Among the characterized glycolipids, MGDG(18:4/16:4) (ion 5418, VIP no 23) is significantly overexpressed in NR (*p* value < 0.05, FDR < 0.05; *t*-test) and most likely corresponds to MGDG(18:4(6Z,9Z,12Z,15Z)/16:4(4Z,7Z,10Z,13Z)) previously identified in methanolic extracts of *T. chui*, which has been shown to exhibit NO inhibitory activity (Banskota et al., 2013). NO is a gas with a signalling function in various mammalian tissues, allowing it to regulate many physiological processes, but is also responsible for several diseases (Lundberg and Weitzberg, 2022). In plants, NO plays various roles in the regulation of hormonal responses, maturation, senescence, germination, root development, stomatal function, pathogen defence mechanisms and apoptosis (Beligni and Lamattina, 2001; del Río et al., 2004; Wendehenne et al., 2004). In bacteria, NO is involved in both formation and dispersion of biofilm (Cutruzzolà and Frankenberg-Dinkel, 2016). While research on microalgae remains limited, existing evidence suggests a regulatory role for NO in N uptake and in the biosynthesis of specific metabolites (Astier et al., 2021).

In plant, NO production is mediated by NO synthases and NO_3_^−^ reductases (Beligni and Lamattina, 2001; del Río et al., 2004; Wendehenne et al., 2004). NO_3_^−^ reductases activity promoting NO production also exists in microalgae (Astier et al., 2021; Mallick et al., 2000; Sakihama et al., 2002). Mallick et al. (2000) (Mallick et al., 2000) have shown that high concentrations of NO_3_^−^ positively influence NO levels.

NO appears to play a role in microalgal adhesion (Thompson et al., 2008; Vardi et al., 2008; Leflaive and Ten-Hage, 2011a; Allen et al., 2015). For example, Thompson et al. (2008) (Thompson et al., 2008) showed that intracellular NO concentrations in the diatom *Seminavis robusta* F3–61B are four-fold higher when cells are exposed to surfaces poorly suited for adhesion, such as hydrophilic surfaces – like the acid-washed glass used in our experiments. Artificially increasing intracellular NO levels in *S. robusta* F3–61B was also associated with a reduction in the ability of this species to adhere (Thompson et al., 2008). Similarly, Vardi et al. (2008) (Vardi et al., 2008) demonstrated that overexpression of the PtNOA gene in the diatom *Phaeodactylum tricornutum* resulted in increased NO production and decreased adhesion. A similar inhibition of adhesion linked to elevated intracellular NO levels was also observed in the diatom *Fistulifera saprophila* (Allen et al., 2015).

**Fig. 10:**
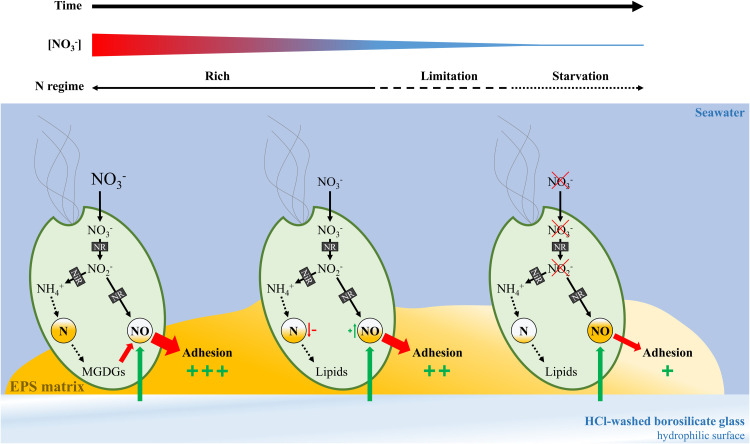
**Hypothetical feedback loop for phototrophic biofilm adhesion regulated by nitric oxide.** Schematic of the proposed adhesion mechanism in *Tetraselmis suecica* AC254 biofilm involving nitric oxide (NO), modulated by nitrate (NO_3_^−^) availability. Abbreviations: nitrite (NO_2_^−^); nitrate reductase (NR); nitrite reductase (NiR); extracellular polymeric substances (EPS); monogalactosyldiacylglycerols (MGDGs); and hydrochloric acid (HCl).

These results lead us to hypothesise that the NO_3_^−^-rich conditions in our experiment, combined with the hydrophilic properties of acid-washed borosilicate glass, enhanced cellular NO production in the biofilms, likely through the activity of NO_3_^−^ reductase. In parallel with, or in response to, this increased NO concentration, NO-inhibiting MGDGs may be synthesised to counteract NO-mediated adhesion loss. Moreover, our results suggest that biosynthesis of NO-inhibiting MGDGs ceases in NO_3_^−^-limited *T. suecica* biofilms. We hypothesise that in these NO_3_^−^-limited conditions, intracellular NO concentration remains above a critical threshold, impairing cell adhesion (Fig. 10). This could explain the relative increase in the F-TL fraction at the expense of the F-DL fraction observed in NL biofilms, in the absence of any visible detachment (Fig. 6).

Chemical mediation mechanisms involving NO in microalgae have also been suggested. Previous studies have shown that 2E,4E-decadienal, an aldehyde produced by diatoms, induces NO production and reduces diatom adhesion (Vardi et al., 2008; Leflaive and Ten-Hage, 2011a, 2011b). Similarly, unidentified compounds from the filamentous alga *Uronema confervicolum*, suspected to act as infochemicals, elicit comparable responses in *F. saprophila* (Allen et al., 2015). These results suggest that the formation of phototrophic biofilms may involve chemical mediators exchanged among their constituent microorganisms, potentially resulting in fluctuations in NO levels in response to environmental conditions. Further research is required to assess whether enhancing or inhibiting NO production influences the adhesion of monospecific phototrophic biofilms, and whether alternative N sources replicate the effects observed in this study.

A second MGDG, with one fewer double bond on C18 acyl chain – referred to as MGDG(18:3/16:4) (ion 5788, VIP no 34) in our dataset, is also significantly overexpressed in NR (*p* value < 0.01, FDR < 0.01; *t*-test) and likely corresponding to MGDG(18:3(9Z,12Z,15Z)/16:4(4Z,7Z,10Z,13Z)) – also exhibits NO inhibitory activity (Banskota et al., 2013). According to these authors, increasing unsaturation of C chains in MGDGs enhance NO inhibitory activity, suggesting that other MGDGs identified may exhibit similar property. However, specific studies are needed to confirm this hypothesis. Finally, the ecological function(s) of these glycolipids within biofilm remain unclear. Although MGDGs and DGDGs have been associated with various bioactivities (Alves et al., 2020), none have been documented in *T. suecica* to our knowledge. Further research is necessary to explore the potential bioactive properties of these molecules.

## 4 Conclusion

In conclusion, our study demonstrates a slowdown in protein synthesis and an accumulation of lipids and carbohydrates, strongly suspected in the form of starch, in N-limited biofilms of the chlorophyte *Tetraselmis suecica* AC254. Lipid composition is also modified, notably by a decrease in glycolipids, particularly MGDGs, in favour of others, as yet unidentified, lipids. Among them, MGDG(18:3(9Z,12Z,15Z)/16:4(4Z,7Z,10Z,13Z)), which is abundantly detected only in N-replete biofilms, has previously been reported to inhibit NO synthesis. NO is a signalling molecule involved in numerous regulatory and biosynthetic pathways, and its production is enhanced by the reduction of NO_3_^−^ and NO_2_^−^. In microalgae, intracellular NO content has been correlated with adhesion capacity. We propose a hypothetical feedback loop in which NO_3_^−^ availability regulates NO levels and MGDG biosynthesis, which in turn influence adhesion. This model could provide a mechanistic explanation for the observed differences in adhesion behaviour between N-limited and N-replete *T. suecica* biofilms.

## Funding sources

This work was supported by the Photobiofilm Explorer project, funded by the French National Research Agency (ANR-20-CE43–0008).

## CRediT authorship contribution statement

**Julien Lopez:** Conceptualization, Data curation, Formal analysis, Investigation, Methodology, Validation, Visualization, Writing – original draft. **Amélie Talec:** Investigation, Resources. **Stéphane Greff:** Data curation, Investigation, Resources. **Andrea Fanesi:** Investigation, Resources. **Beat Gasser:** Formal analysis, Investigation, Resources. **Emna Krichen:** Methodology. **Olivier Bernard:** Funding acquisition, Supervision, Project administration. **Antoine Sciandra:** Conceptualization, Funding acquisition, Methodology, Project administration, Resources, Supervision, Writing – original draft.

## Declaration of competing interest

The authors declare the following financial interests/personal relationships which may be considered as potential competing interests:

Olivier Bernard reports financial support was provided by French National Research Agency. If there are other authors, they declare that they have no known competing financial interests or personal relationships that could have appeared to influence the work reported in this paper.

## Appendix Supplementary materials

Image, application 1

Image, application 2

Image, application 3

Image, application 4
