# Supplementary material for: Effects of nitrate limitation on the metabolome of Tetraselmis suecica biofilms
Source: Curr Res Microb Sci. 2025 Oct 30;9:100501. doi: 10.1016/j.crmicr.2025.100501 (PMC12639569; doi:10.1016/j.crmicr.2025.100501)
Supplement: Supplementary file 1 [file mmc1.docx]

AntheraX: Antheraxanthin

ATR-FTIR: Attenuated Total Reflectance Fourier Transform Infrared Spectroscopy

BIOF: Biofilm

C: Carbon

CV: Coefficient of Variation

DGDGs: Digalactosyldiacylglycerols

EPS: Extracellular Polymeric Substances

FAs: Fatty Acids

FDR: False Discovery Rate

FULL: Entire culture

F-DL: Fraction – Deep Layer

F-L1: Fraction – Layer n°1

F-L2: Fraction – Layer n°2

F-L3: Fraction – Layer n°3

F-TL: Fraction – Top Layer

F-NA: Fraction – Non-Adhered cell

HCl: Hydrochloric acid

HPLC: High-Performance Liquid Chromatography

LC-MS: Liquid Chromatography – Mass Spectrometry

MeOH: Methanol

MGDGs: Monogalactosyldiacylglycerols

MUFAs: Monounsaturated Fatty Acids

N: Nitrogen

NeoX: Neoxanthin

NL: Nitrogen-Limited condition

NO: Nitric Oxide

NO_3_^-^: Nitrate

NR: Nitrogen-Replete condition

PCA: Principal Component Analysis

Pheo_*a*: Pheophytin *a*

PLS-DA: Partial Least Squares Discriminant Analysis

PO_4_^3-^: Phosphate

PUFAs: Polyunsaturated Fatty Acids

QC: Quality Control

SFAs: Saturated Fatty Acids

TChl_*a*: Chlorophyll *a*

TChl_*b*: Chlorophyll *b*

UHPLC-ESI(+)-QToF-HRMS/MS:

Ultra High Liquid Chromatography – Electrospray Ionization (positive mode) – Quadrupole Time-of-Flight – High Resolution Mass Spectrometry Tandem

ViolaX: Violaxanthin

VIPs: Variables Importance in Projection

ZeaX: Zeaxanthin
