# Supplementary material for: Effects of nitrate limitation on the metabolome of Tetraselmis suecica biofilms
Source: Curr Res Microb Sci. 2025 Oct 30;9:100501. doi: 10.1016/j.crmicr.2025.100501 (PMC12639569; doi:10.1016/j.crmicr.2025.100501)
Supplement: Supplementary file 2 [file mmc2.docx]

**Supplementary figures** “Effects of nitrate limitation on the metabolome of *Tetraselmis suecica* biofilms”

**
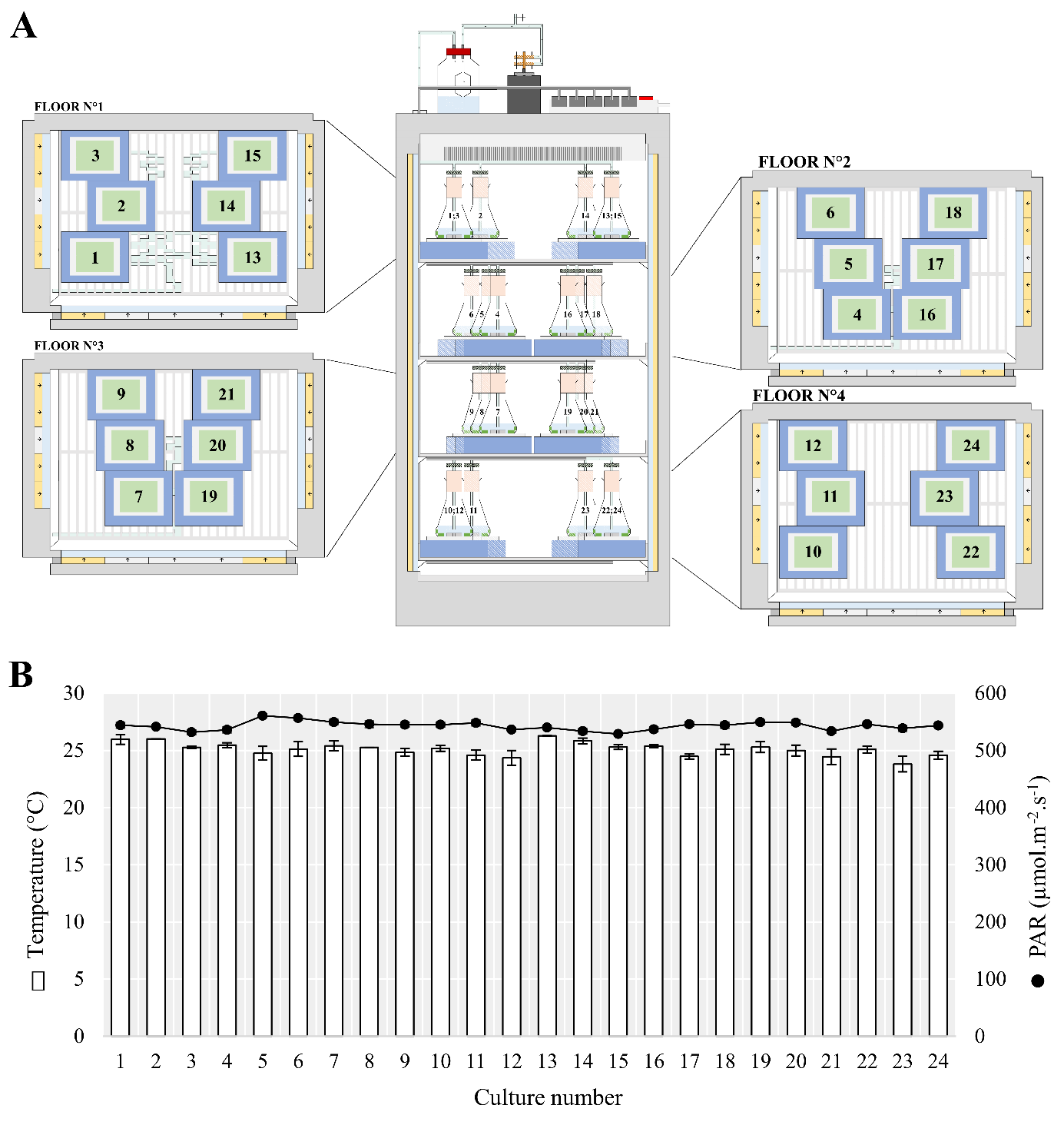
**

***Supplementary figure S1.* Environmental conditions in the incubation chamber for *Tetraselmis suecica* biofilms*.*** A) Schematic representation of the optimized biofilm culture system within a SANYO MLR-351 incubation chamber. B) Temperature (°C; white bars) and photosynthetically active radiation (PAR; µmol.m^-2^.s^-1^; black bars) shown as a function of position inside the chamber.

***
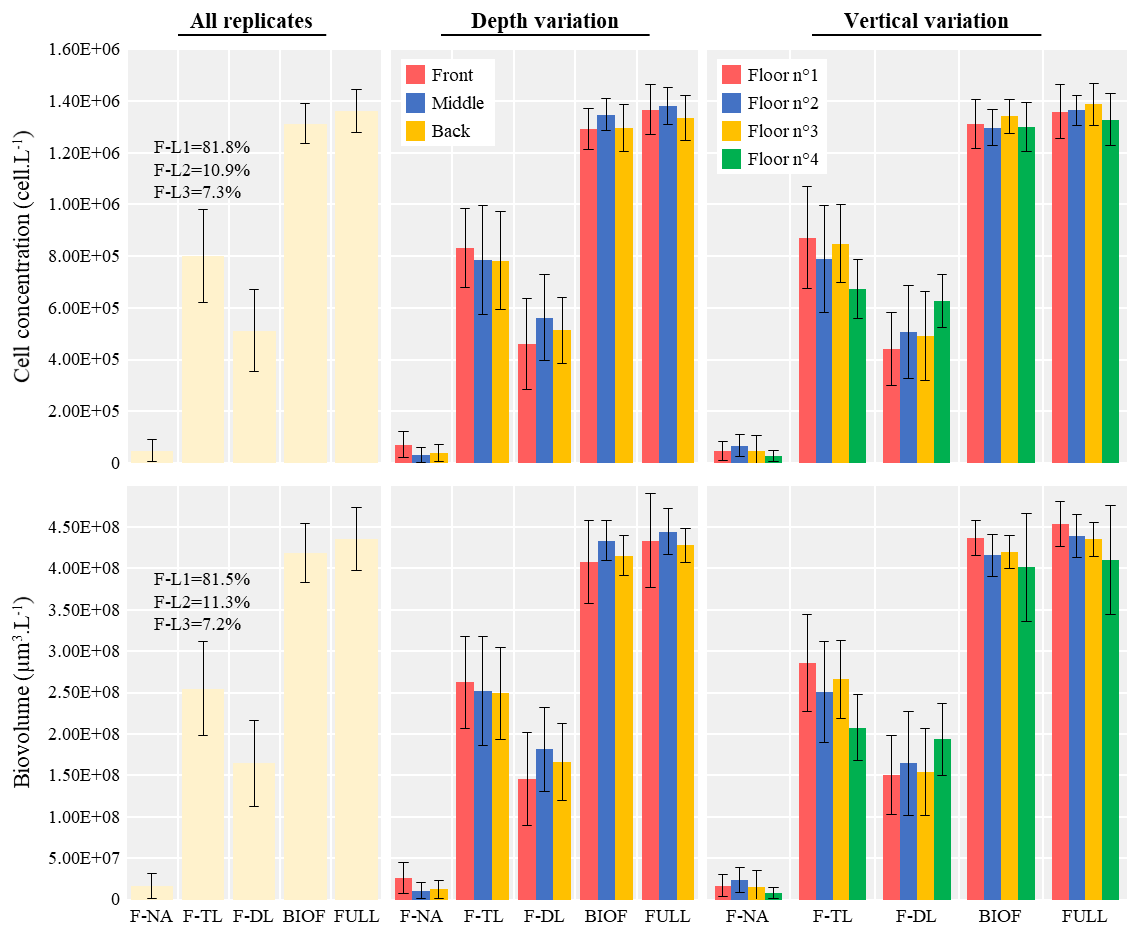
***

***Supplementary figure S2.* Cell concentrations in *Tetraselmis suecica* biofilm fractions after 7 days of incubation.** Cell concentrations measured after 7 days (t7) in fractions, as a function of depth and height within the culture chamber. Fractions: non-adherent cells (F-NA), top layer (F-TL), deep layer (F-DL), biofilm (BIOF = F-TL + F-DL), and all fractions combined (FULL = F-NA + F-TL + F-DL). The proportion of the subfractions F-L1, F-L2 and F-L3 is indicated above the fraction F-TL. Each bar represents the mean of three biological replicates; error bars indicate standard deviations.

***
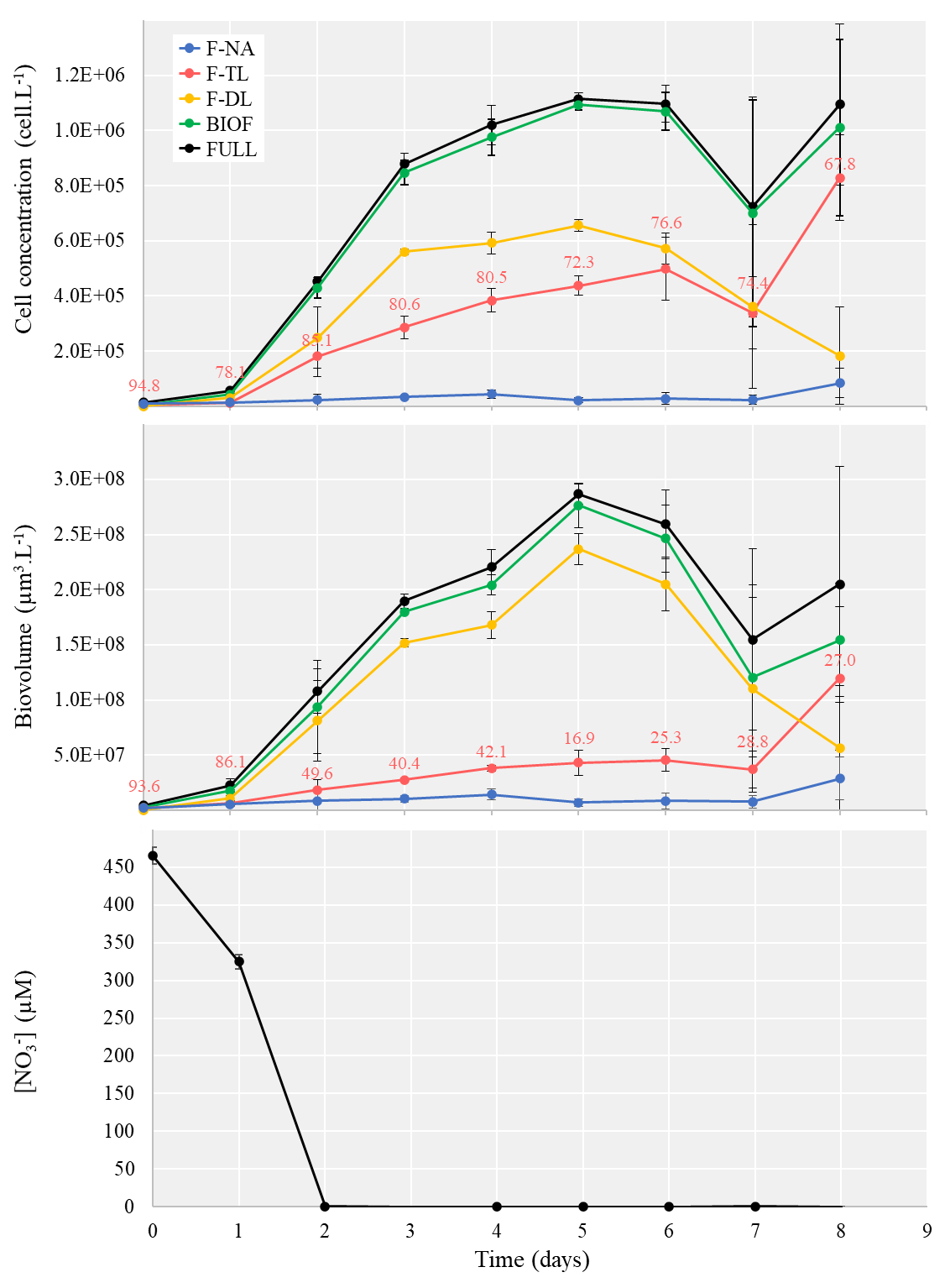
***

***Supplementary figure S3.* Temporal variations in cell concentration, biovolume, and nitrate levels in *Tetraselmis suecica* biofilms.** Changes in cell concentration (top), biovolume (middle), and residual nitrate concentration (bottom) across different fractions of biofilm over 8 days of incubation. Fractions: non-adherent cells (F-NA), top layer (F-TL), deep layer (F-DL), biofilm (BIOF = F-TL + F-DL), and all fractions combined (FULL = F-NA + F-TL + F-DL). The proportion of the subfraction F-L1 is indicated in red above the fraction F-TL. Each point represents the mean of three independent cultures; vertical bars indicate standard deviations.

**
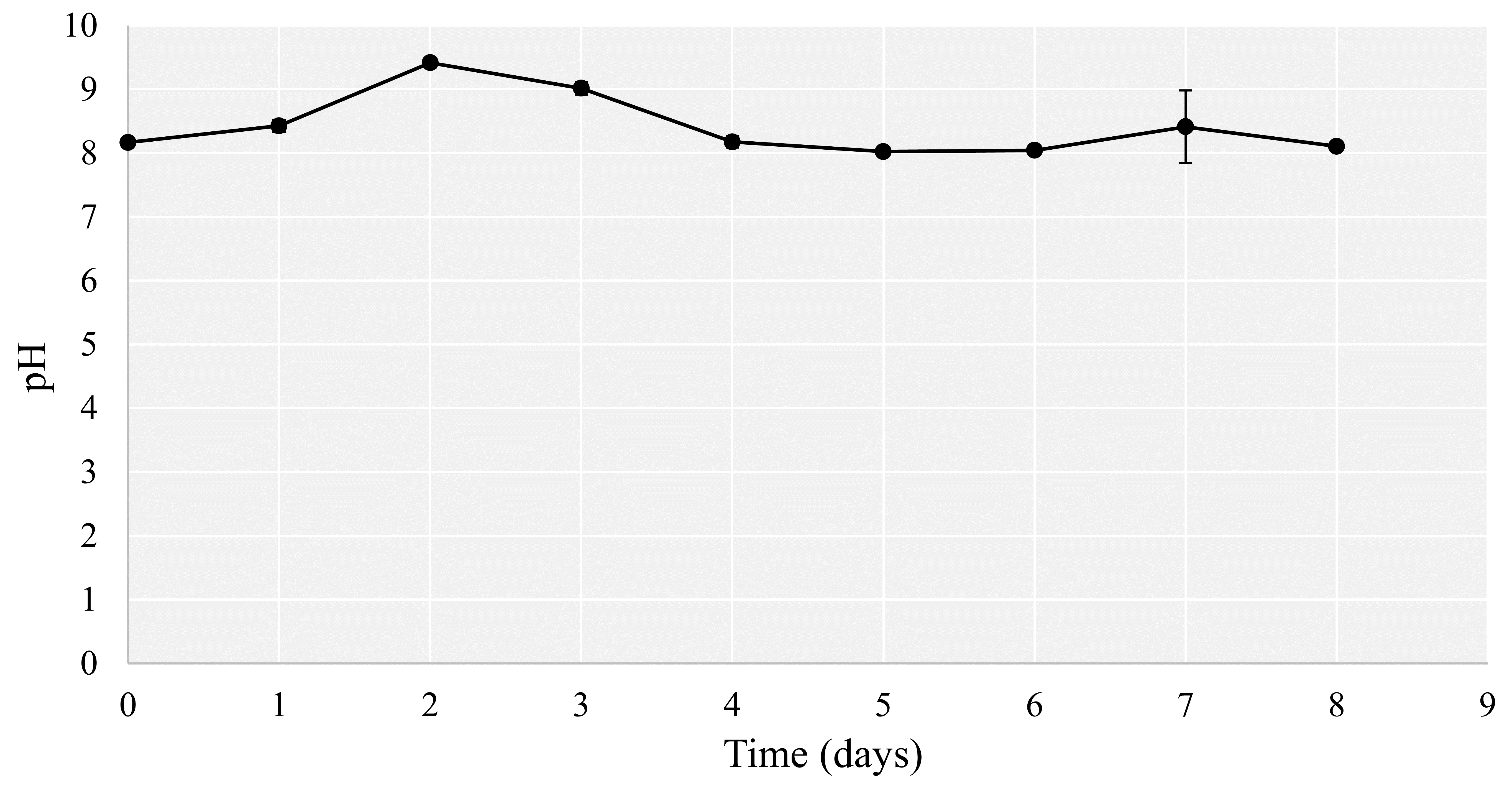
**

***Supplementary figure S4.*** **Temporal variation in pH of the supernatant in *Tetraselmis suecica* biofilm cultures.** pH variation in the supernatant (F-NA fraction) of biofilm cultures over 8 days of incubation. Each point represents the mean of three independent cultures; vertical bars indicate standard deviations.

**
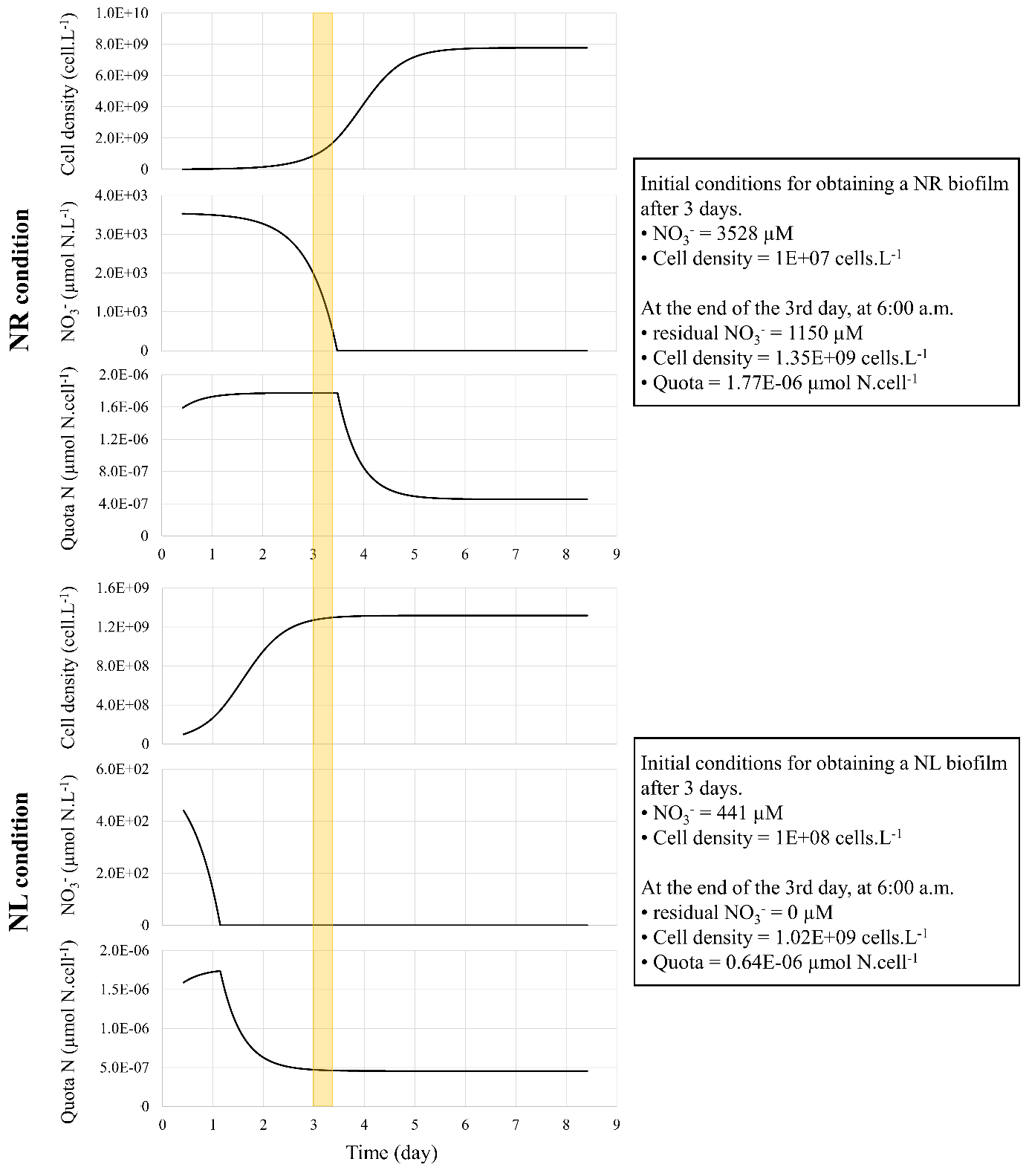
**

***Supplementary figure S5.*** **Droop model-based simulation used to determine the initial conditions and optimal sampling time of *Tetraselmis suecica* biofilms.** Simulation of optimal sampling conditions for nitrogen-replete (NR; up graph) and nitrogen-limited (NL; down graph) biofilms using the Droop model. The yellow band indicates the sampling window meeting the defined criteria, corresponding to three days of incubation under the initial experimental conditions shown in the boxes on the right.

***Supplementary figure S6.* Summary of the metabolomics data filtering workflow.** For more details of the workflow use, see the following tabs in the Supplementary LCMS table: (0) Processing, (1) Table, and (3) MetaboAnalyst_Preview.

**
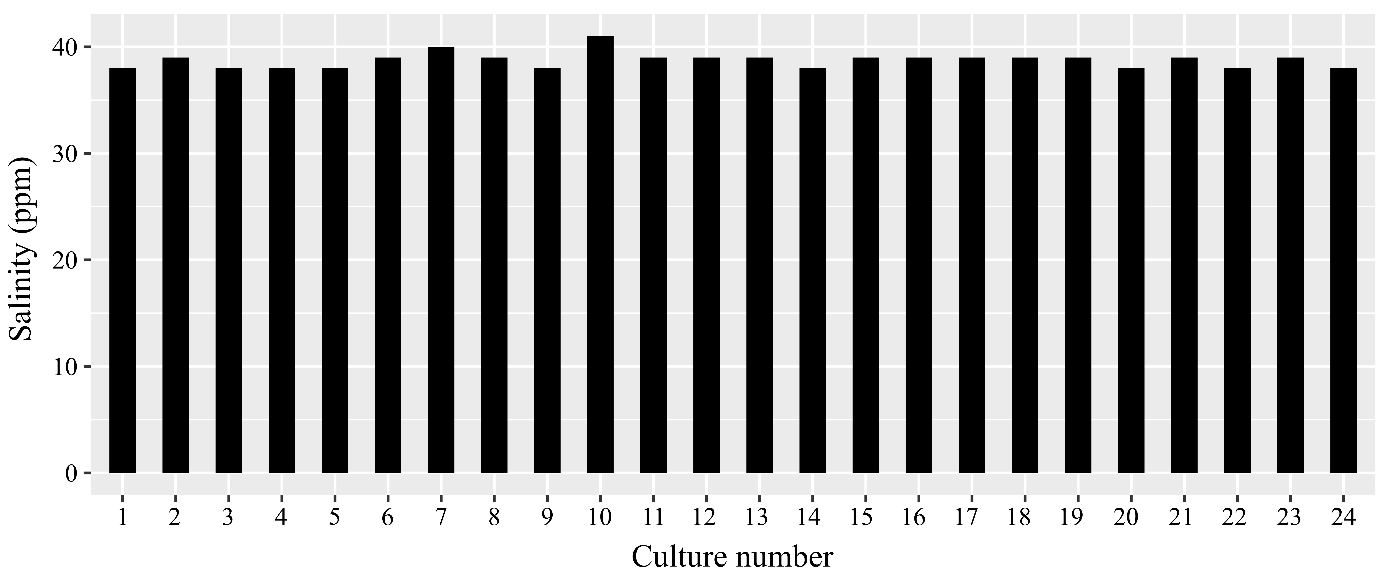
**

***Supplementary figure S7.*** **Salinity in the supernatant of *Tetraselmis suecica* cultures.** Salinity measured in the supernatant (F-NA fraction) of cultures and corresponding blanks after three days of incubation.

**
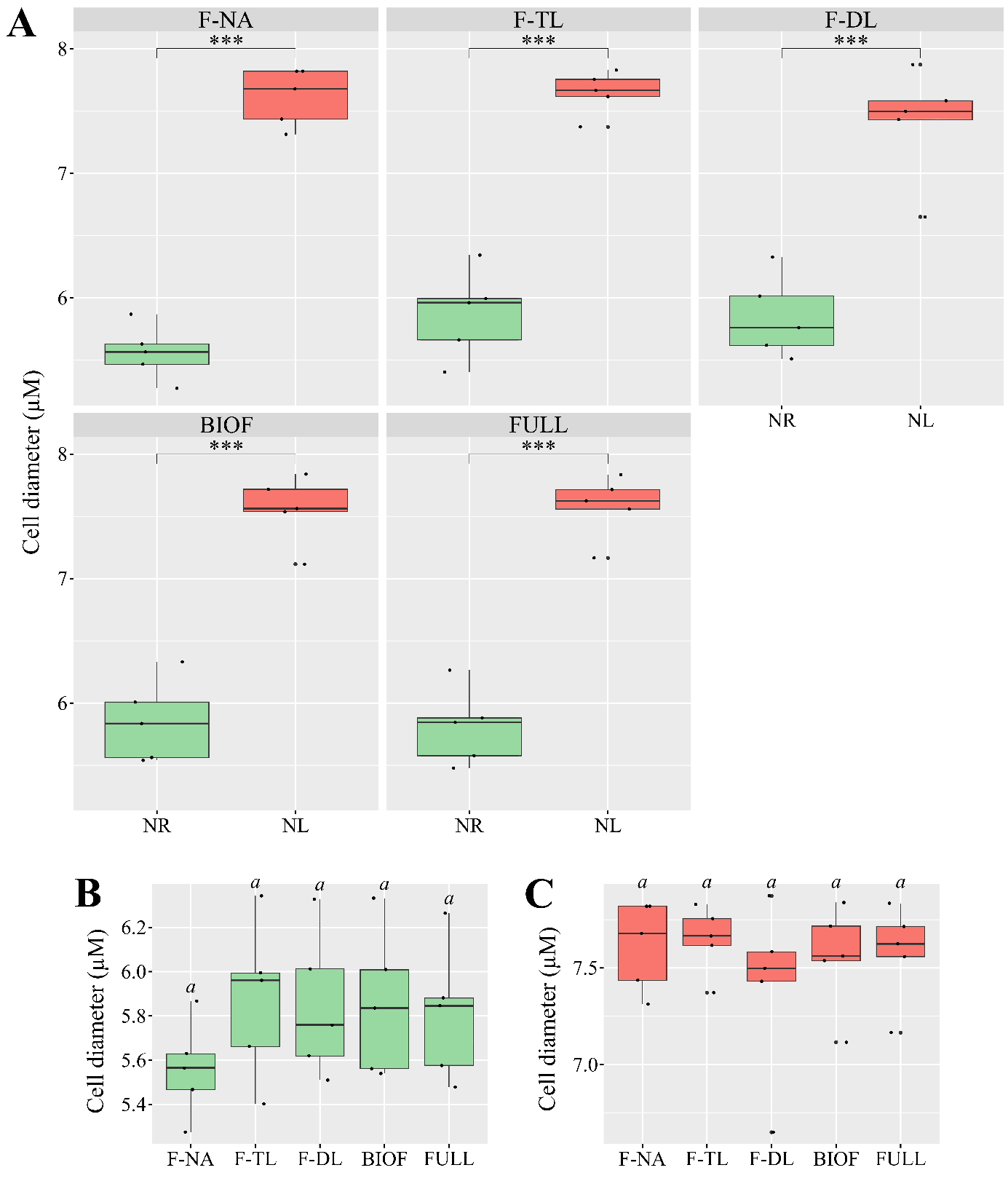
**

***Supplementary figure S8.*** **Cell diameter** **across *Tetraselmis suecica* biofilm.** Cell diameter measured in nitrogen-replete (NR; green) and nitrogen-limited (NL; red) biofilm and culture fractions (A). ***: p-value < 0.001. Cell concentrations in NR (B) and NL (C) culture fractions. Fractions: non-adherent cells (F-NA), top layer (F-TL), deep layer (F-DL), biofilm (BIOF = F-TL + F-DL), and all fractions combined (FULL = F-NA + F-TL + F-DL). Statistical differences are indicated by different italic letters above the box plots.

**
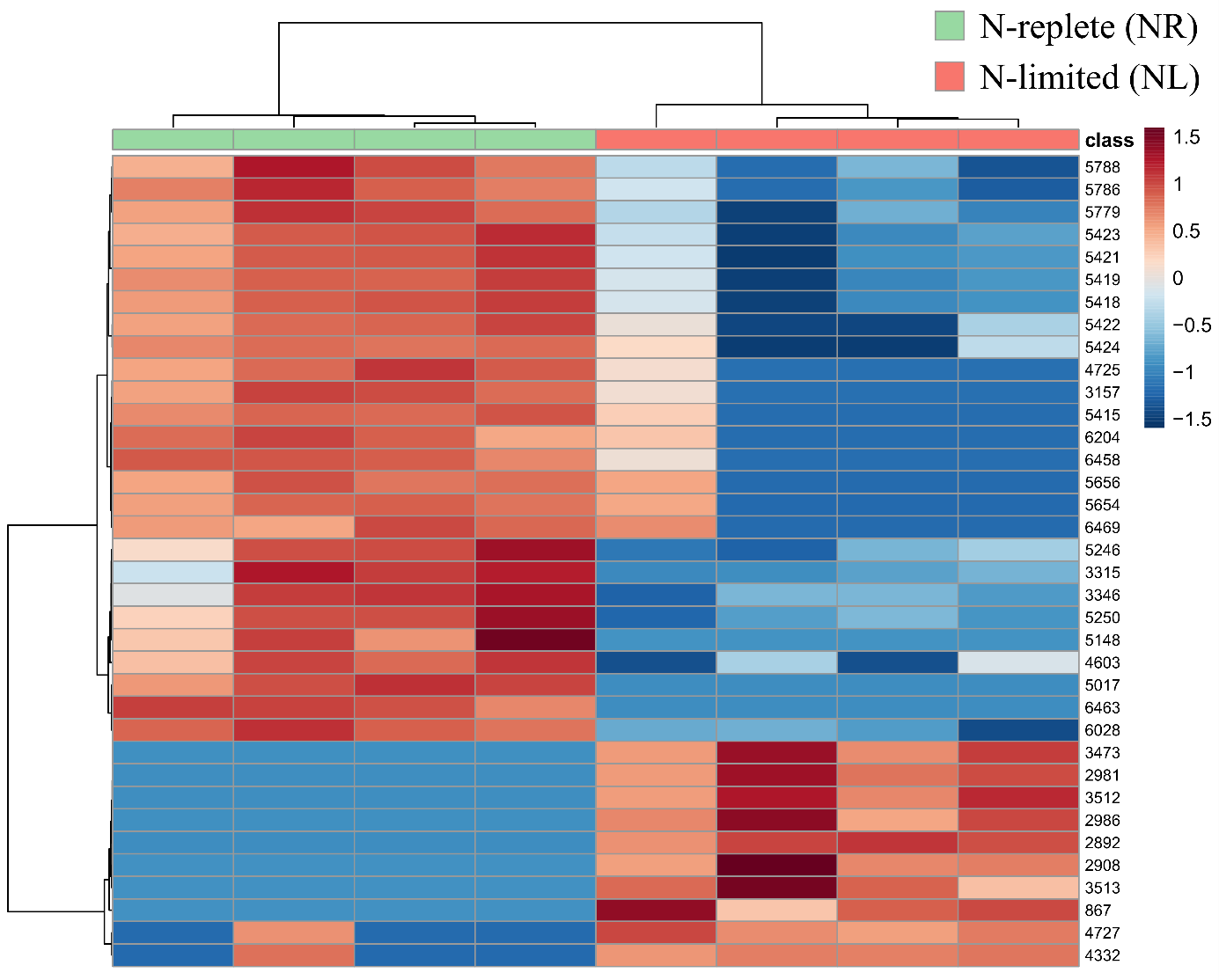
**

***Supplementary figure S9.* Heatmap of discriminant metabolites in *Tetraselmis suecica*.** Heatmap (multivariate analysis) of UHPLC-ESI(+)-QToF-MS data from MeOH extracts of nitrogen-replete (NR; green) and nitrogen-limited (NL; red) biofilms. Only the 36 most discriminant variables (metabolites) identified by the analysis (VIPs) are shown. Red shades indicate relative overexpression; blue shades indicate underexpression. Analysis and figure generated using MetaboAnalyst (v6.0).
