# Supplementary material for: Effects of nitrate limitation on the metabolome of Tetraselmis suecica biofilms
Source: Curr Res Microb Sci. 2025 Oct 30;9:100501. doi: 10.1016/j.crmicr.2025.100501 (PMC12639569; doi:10.1016/j.crmicr.2025.100501)
Supplement: Supplementary file 3 [file mmc3.docx]

**Supplementary material and methods** “Effects of nitrate limitation on the metabolome of *Tetraselmis suecica* biofilms”

# Development of the biofilm culture process

We developed and optimized a dedicated biofilm culture system to reliably investigate the impact of N status on the metabolome of *T. suecica* biofilms while satisfying the criteria required for metabolomic analysis (i.e., environmental control, measurement accuracy, and contamination-free extraction). The culture system was designed to satisfy the following conditions: *(i)* no biological or chemical contamination, *(ii)* low-volume culture format, *(iii)* easy and rapid biofilm harvesting, *(iv)* sufficient replication (≥ 5 replicates per condition), *(v)* control and stability of all growth parameters (temperature, light, pH, nutrients except N), *(vi)* high reproducibility between replicates, and *(vii)* precise control of the N status of microalgae.

To this end, classical planktonic cultures were adapted. Borosilicate glass Erlenmeyer flasks equipped with a PTFE magnetic stir bar were used to support development of contamination-free and significant microalgal biofilm (condition *i*) in small volume (condition *ii*). Moderate rotation speed (250 rpm) promotes cell adhesion to the glass wall, without disrupting the biofilm after its formation. Indeed, the effect of shear stress on biofilm formation had previously been observed in bacteria (Stoodley *et al.*, 1998; Beyenal and Lewandowski, 2002; Moreira *et al.*, 2022; Tsagkari *et al.*, 2022) and the microalga *Chlorella vulgaris* (Fanesi *et al.*, 2021).

This approach avoids the need for additional support materials, thus minimizing contamination risks. The direct addition of organic solvents within flasks enabled both rapid metabolite extraction and reduced contamination during harvesting (conditions *i* and *iii*). However, since metabolite extraction requires complete biofilm destruction, we allocated separate flasks for metabolomic and biochemical analyses (5 replicates per condition). This separation ensured that destructive sampling for one analysis did not compromise data collection for the other (condition *iv*).

This large number of flasks requires a spacious and homogeneous incubation environment. An incubation chamber (MLR-351, SANYO) was selected and configured to provide optimal light intensity (540 µmol quanta·m^-2^·s^-1^) (Montes-González *et al.*, 2021) and temperature (25 °C) (Sas *et al.*, 2021) conditions for *T. suecica* growth. Light intensities measured at the centre of each Erlenmeyer flask using a photometer (UML-500 Universal Light Meter & Data Logger, WALZ) showed good uniformity between positions flasks within the incubation chamber (CV = 0.06; see *Supplementary figure S1A, B*). A slight vertical temperature gradient (23.8 to 26.3 °C) due to convection from fluorescent lights (Lumilux Cool white, L 36W/940, 120 cm, OSRAM) was detected (see *Supplementary figure S1B*), but did not significantly affect growth, as shown in preliminary planktonic tests (see *Supplementary figure S2*).

To prevent evaporation and carbon (C) limitation, a gentle supply of humidified, filtered air was added above the cultures. This approach avoided bubbling directly into the medium, thereby protecting biofilm integrity. These precautions maintained pH below 9.0 and ensured sufficient inorganic C for photosynthesis (condition *v*).

Two preliminary experiments were conducted successively using *T. suecica* cultures (condition *vi*) to assess the reproducibility of biofilm formation. These pre-experiments validate the reproducibility and reliability of the biofilm culture system and developed protocol for both metabolomic and biochemical analyses (see Supplementary Materials and Methods, and Supplementary Figures S1 to S4 for details).

# Determination of the experimental parameters of NR and NL biofilms

One of the main requirements of our metabolomic analysis is that it must be performed on NL and NR biofilms that are inoculated and sampled simultaneously, with sufficient biomass to allow metabolomic or biochemical analyses. Furthermore, it is essential that N status be the only factor responsible for differences in metabolome response. In other words, all other growth factors (nutrients other than N, temperature, light and pH) must either be non-limiting or maintained at equivalent levels between the two experimental conditions. Among these, only temperature is unaffected by biofilm development. The average photon flux through the biofilm decreased as its thickness increased. Here, we assumed that the light conditions in harvested NR and NL biofilms were comparable if the biofilms had similar thickness. Nutrient concentrations declined as they were consumed. For nutrients other than N [i.e. micronutrients and phosphate (PO_4_^3-^)], we assumed that any differences in concentration between the NR and NL conditions did not affect microalgal physiology, provided they remained in excess.

For this, we ensured that the relative concentrations of micronutrients (trace metals, vitamins) were maintained across the different media used during preparation. Although low, these concentrations remained significantly higher than those typically found in the natural environment (Sohrin and Bruland, 2011; Pinedo-Gonzalez *et al.*, 2015). The initial PO_4_^3-^ concentration in the culture media was doubled relative to the Redfield ratio (Redfield, 1934). Similarly, the N:P ratio was carefully controlled to avoid influencing the biosynthesis of various compounds such as pigments or lipids (Song *et al.*, 2016).

In order to define the initial experimental conditions for cell density and NO_3_^-^ concentration that, over an identical timeframe, would result in adequately concentrated cultures exhibiting distinct N statuses, we opted to base our approach on the outcomes of a growth model rather than undertake preliminary experiments that would require considerable time. The Droop quota model (Droop, 1968) offers the advantage of explicitly accounting for the N status of the cell. The central assumption of this model is that growth rate *µ* (d^-1^) depends on the amount of limiting nutrient present within the cell – here, total cellular N – referred to as the internal quota *q* (µmol N·cell^-1^), according to the following expression (1).

(1) $\mu\left( q \right)=\bar{\mu} \left( 1-{q_{0}}/q \right)$

where *q_0_* is the minimum subsistence N quota below which growth can no longer occur, and $\bar{\mu}$ is the theoretical maximum growth rate achieved when the internal quota *q* is infinite. The uptake rate *ρ* (µmol N·cell^-1^·d^-1^) of NO_3_^-^ at a concentration *s* (µmol·L^-1^) is described by the Michaelis-Menten equation (2).

(2) $\rho\left( s \right)=\rho_{m}\left( \frac{s}{s+k_{s}} \right)$

where *ρ_m_* is the maximum uptake rate and *k_s_* is the half-saturation constant. Accordingly, the dynamics of cell density *x* (cell·L^-1^) (3), NO_3_^-^ concentration *s* (µmol N·L^-1^) (4), and internal N quota *q* (µmol N·cell^-1^) (5) are described as follows:

(3) ${dx}/{dt}=\mu\left( q \right).x$

(4) ${ds}/{dt}= -\rho\left( s \right).x$

(5) ${dq}/{dt}= \rho\left( s \right)-\mu\left( q \right).q$

This system of equations was numerically integrated using the MATLAB function ODE23. Parameter values (**Table 0**) were obtained by fitting the model – using OPTIMSET function – to experimental data from planktonic cultures of *T. suecica* grown under light and temperature conditions similar to those used in our study.

**Table 0.** Droop model parameters estimated by fitting the model to experimental data.

| Parameter | Value | Unit |
| --- | --- | --- |
| *ρ_m_* | 3.09E-06 | µmol N·cell^-1^·d^-1^ |
| *k_s_* | 0.52 | µmol N |
| $\bar{\mu}$ | 2.34 | d^-1^ |
| *q_0_* | 4.55E-07 | µmol N·cell^-1^ |

This simulation shows that a batch culture gradually progresses through three distinct phases: an initial phase of maximum growth (strictly exponential) as long as residual NO_3_^-^ is available; a subsequent phase of limited growth while the internal N quota remains above the subsistence threshold; and finally, a phase of zero growth once the quota reaches its minimum value (*q₀*). The N-deficient state can be considered as a condition of pronounced limitation, although the boundary between limitation and deficiency is not sharply defined. The simulation also demonstrates that the true N status of a culture cannot be inferred solely from the presence or absence of NO_3_^-^ in the medium. The only truly relevant indicator is the direct measurement of the internal N quota. Assuming that the model – originally validated for planktonic cultures – predicts similar growth dynamics in biofilm systems, we used it to determine the initial NO_3_^-^ and cell density conditions, as well as the appropriate culture duration, to ensure that all specified conditions were satisfied (condition *vii*; *see Supplementary Figure S5*).

# References

Beyenal, H., Lewandowski, Z. 2002. Internal and external mass transfer in biofilms grown at various flow velocities. Biotechnology Progress, 18(1), 55-61. https://doi.org/10.1021/bp010129s

Droop, M.R. 1968. Vitamin B12 and Marine Ecology. IV. The Kinetics of Uptake, Growth and Inhibition in Monochrysis Lutheri. Journal of the Marine Biological Association of the United Kingdom, 48(3), 689-733. https://doi.org/10.1017/S0025315400019238

Fanesi, A., Lavayssière, M., Breton, C., Bernard, O., Briandet, R., Lopes, F. 2021. Shear stress affects the architecture and cohesion of Chlorella vulgaris biofilms. Scientific Reports, 11(1), 4002. https://doi.org/10.1038/s41598-021-83523-3

Montes-González, O., Gonzalez-Silvera, A., Valenzuela-Espinoza, E., Santamaría-del-Angel, E., Lopez-Calderon, J. 2021. Effect of light intensity and nutrient concentration on growth and pigments of the green microalga Tetraselmis suecica. Latin American Journal of Aquatic Research, 49, 431-441. https://doi.org/10.3856/vol49-issue3-fulltext-2632

Moreira, J., Vaz, B., Barcelos Cardias, B., Gonzales Cruz, C., Almeida, A., Costa, J.A., Morais, M. 2022. Microalgae Polysaccharides: An Alternative Source for Food Production and Sustainable Agriculture. Polysaccharides, 3, 441-457. https://doi.org/10.3390/polysaccharides3020027

Pinedo-Gonzalez, P., West, A.J., Tovar-Sanchez, A., Duarte, C., Maranon, E., Cermeño, P., González-Benítez, N., *et al.* 2015. Surface distribution of dissolved trace metals in the oligotrophic ocean and their influence on phytoplankton biomass and productivity. Global Biogeochemical Cycles, 29, n/a-n/a. https://doi.org/10.1002/2015GB005149

Redfield, A.C. 1934. On the proportions of organic derivatives in sea water and their relation to the composition of plankton. *In* James Johnstone memorial volume. p. 156.

Sas, A., Turki, A., Affan, M., Al-Taisan, W., Das, S.K., Su, S., Cob, Z. 2021. The influence of temperature and nutrient concentrations on growth rate, biomass, Chlorophyll-a, and biochemical compositions of Tetraselmis suecica (Chlorophyta). IOP Conference Series: Earth and Environmental Science, 880, 012014. https://doi.org/10.1088/1755-1315/880/1/012014

Sohrin, Y., Bruland, K.W. 2011. Global status of trace elements in the ocean. TrAC Trends in Analytical Chemistry, 30(8), 1291-1307. https://doi.org/10.1016/j.trac.2011.03.006

Song, D., Xi, B., Sun, J. 2016. Characterization of the growth, chlorophyll content and lipid accumulation in a marine microalgae Dunaliella tertiolecta under different nitrogen to phosphorus ratios. Journal of Ocean University of China, 15(1), 124-130. https://doi.org/10.1007/s11802-016-2797-z

Stoodley, P., Dodds, I., Boyle, J.D., Lappin-Scott, H.M. 1998. Influence of hydrodynamics and nutrients on biofilm structure. Journal of Applied Microbiology, 85 Suppl 1, 19S-28S. https://doi.org/10.1111/j.1365-2672.1998.tb05279.x

Tsagkari, E., Connelly, S., Liu, Z., McBride, A., Sloan, W.T. 2022. The role of shear dynamics in biofilm formation. NPJ biofilms and microbiomes, 8(1), 33. https://doi.org/10.1038/s41522-022-00300-4
